# A chromosome‐anchored genome assembly for Lake Trout (*Salvelinus namaycush*)

**DOI:** 10.1111/1755-0998.13483

**Published:** 2021-08-14

**Authors:** Seth R. Smith, Eric Normandeau, Haig Djambazian, Pubudu M. Nawarathna, Pierre Berube, Andrew M. Muir, Jiannis Ragoussis, Chantelle M. Penney, Kim T. Scribner, Gordon Luikart, Chris C. Wilson, Louis Bernatchez

**Affiliations:** ^1^ Department of Integrative Biology Michigan State University East Lansing MI USA; ^2^ Ecology, Evolution, and Behavior Program Michigan State University East Lansing MI USA; ^3^ Institut de Biologie Intégrative et des Systèmes Université Laval Quebec QC Canada; ^4^ McGill Genome Centre Department of Human Genetics Montreal QC Canada; ^5^ Department of Human Genetics Canadian Centre for Computational Genomics (C3G McGill University Montréal QC Canada; ^6^ Great Lakes Fishery Commission Ann Arbor MI USA; ^7^ Environmental and Life Sciences Graduate Program Trent University Peterborough ON Canada; ^8^ Department of Fisheries and Wildlife Michigan State University East Lansing MI USA; ^9^ Fish and Wildlife Genomics Group University of Montana Missoula MT USA; ^10^ Flathead Lake Biological Station Division of Biological Sciences University of Montana Polson MT USA; ^11^ Aquatic Research and Monitoring Section Ontario Ministry of Natural Resources and Forestry Peterborough ON Canada

**Keywords:** genome assembly, genomics, Lake Trout, salmonid, *Salvelinus*

## Abstract

Here, we present an annotated, chromosome‐anchored, genome assembly for Lake Trout (*Salvelinus namaycush*) – a highly diverse salmonid species of notable conservation concern and an excellent model for research on adaptation and speciation. We leveraged Pacific Biosciences long‐read sequencing, paired‐end Illumina sequencing, proximity ligation (Hi‐C) sequencing, and a previously published linkage map to produce a highly contiguous assembly composed of 7378 contigs (contig N50 = 1.8 Mb) assigned to 4120 scaffolds (scaffold N50 = 44.975 Mb). Long read sequencing data were generated using DNA from a female double haploid individual. 84.7% of the genome was assigned to 42 chromosome‐sized scaffolds and 93.2% of Benchmarking Universal Single Copy Orthologues were recovered, putting this assembly on par with the best currently available salmonid genomes. Estimates of genome size based on k‐mer frequency analysis were highly similar to the total size of the finished genome, suggesting that the entirety of the genome was recovered. A mitochondrial genome assembly was also produced. Self‐versus‐self synteny analysis allowed us to identify homeologs resulting from the salmonid specific autotetraploid event (Ss4R) as well as regions exhibiting delayed rediploidization. Alignment with three other salmonid genomes and the Northern Pike (*Esox lucius*) genome also allowed us to identify homologous chromosomes in related taxa. We also generated multiple resources useful for future genomic research on Lake Trout, including a repeat library and a sex‐averaged recombination map. A novel RNA sequencing data set for liver tissue was also generated in order to produce a publicly available set of annotations for 49,668 genes and pseudogenes. Potential applications of these resources to population genetics and the conservation of native populations are discussed.

## 1 INTRODUCTION

**FIGURE 1:**
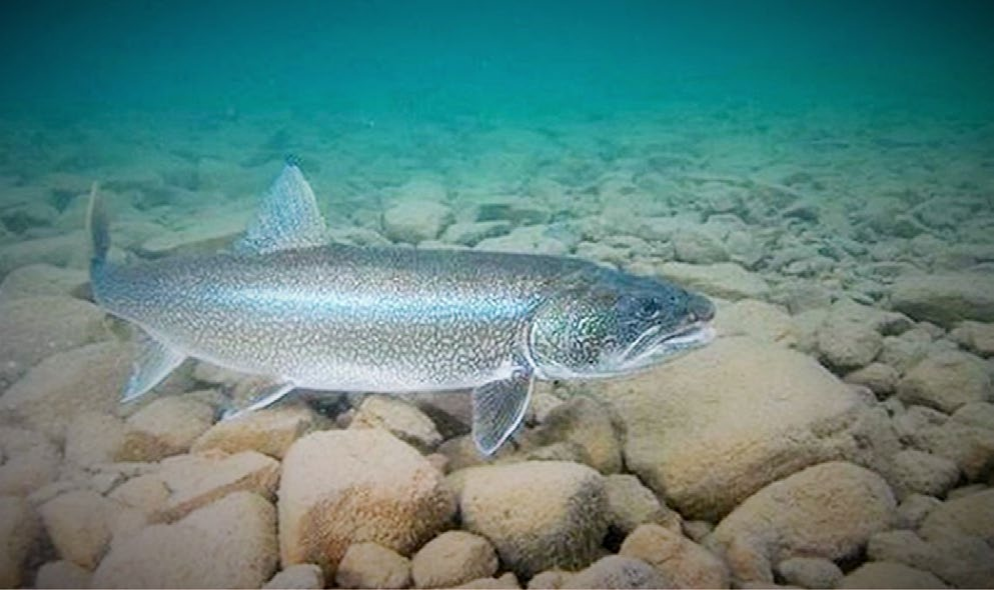
Photograph of an adult Lake Trout (*Salvelinus namaycush*) from Great Bear Lake, Northwest Territories, Canada. Photo credit: Andrew Muir

Key questions in evolution and conservation biology can only be addressed using genomic approaches and appropriate study species. Lake Trout (*Salvelinus namaycush*; Figure 1) are a top predator in many lentic ecosystems across northern North America and express exceptional levels of ecotypic variation (Muir et al., 2014, 2016), making them an ideal study species for exploring the processes of ecological speciation and adaptive diversification. The post‐Pleistocene parallel evolution of diverse Lake Trout ecotypes has been likened to the adaptive radiation of cichlid species in the Great Lakes of east Africa (Muir et al., 2016); however, the radiation of Lake Trout ecotypes appears to have occurred over a relatively short evolutionary timescale (Harris et al., 2015, ~8000 years). At least three distinct Lake Trout ecotypes (lean, siscowet, and humper) once existed throughout the Laurentian Great Lakes (Hansen, 1999) and anecdotal evidence suggests that as many as 10 easily differentiable forms once existed in Lake Superior (Goodier, 1981). High levels of ecotypic variation have also been documented in contemporary populations across the species range (Blackie et al., 2003; Chavarie et al., 2015; Hansen et al., 2016; Zimmerman et al., 2006), with as many as five trophic ecotypes being found in a single lake (Marin et al., 2016).

Lake Trout are also ancestrally autotetraploid, with the common ancestor of all salmonids having undergone a whole genome duplication event (WGD) approximately 80–100 million years ago (Berthelot et al., 2014; Lien et al., 2016; Macqueen & Johnston, 2014). For this reason, salmonids have long been considered ideal study species for understanding the evolutionary consequences of WGD (Allendorf & Thorgaard, 1984; Ohno, 1970). Previous studies have demonstrated that salmonid genomes exhibit a mixture of disomic and tetrasomic inheritance (Allendorf & Danzmann, 1997) and have suggested that salmonid homeologs can be partitioned into two broad categories – ancestral ohnologue resolution regions (AORe) and lineage specific ohnologue resolution regions (LORe; Robertson et al., 2017). AORe regions exhibit elevated differentiation between homeologs because these regions returned to a state of disomic inheritance prior to the radiation of salmonid species. Conversely, LORe regions are characterized by extremely low levels of sequence differentiation between homeologs due to delayed rediploidization. Given the high levels of ecotypic diversity observed in Lake Trout, and the potential for WGD to facilitate the evolution of novel phenotypes (Ohno, 1970; Macqueen & Johnston, 2014; Van de Peer et al., 2017) and reproductive isolation (Lynch & Force, 2000), research exploring the genetic basis for ecotypic differentiation and incipient speciation in Lake Trout could provide important insights about the role of LORe and AORe regions in more recent adaptive radiations.

Furthermore, many Lake Trout populations, particularly those in the Laurentian Great Lakes, have been severely reduced in abundance or distribution, or extirpated, due to invasive species introductions and overfishing (Smith, 1968). Following the basin‐wide collapse of the lake whitefish (*Coregonus clupeaformis*) commercial fishery in the Great Lakes during the early 20th century, fishing pressure was transferred to Lake Trout populations, which partially contributed to population declines starting in the 1930s (Hansen, 1999). A novel predator, the sea lamprey (*Petromyzon marinus*), also invaded the Great Lakes during this time, leading to further increases in adult Lake Trout mortality and functional extirpation from all lakes except Lake Superior and a small, isolated, population in Lake Huron (Hansen, 1999). The restoration program that commenced largely focused on reducing sea lamprey predation, reducing fishing pressure, creating aquatic refuges, and stocking juvenile Lake Trout from a diverse collection of domesticated strains originating from multiple source populations (Hansen, 1999; Krueger et al., 1983). Lake Trout populations in Lake Superior rebounded relatively quickly; however, the re‐emergence of natural reproduction in other lakes was hindered by high levels of lamprey predation on adult Lake Trout (Pycha, 1980), predation on juveniles by invasive alewife (Madenjian et al., 2008), reduced juvenile survival caused by thiamine deficiency (Fitzsimons et al., 2009), and potentially reduced hatching success associated with PCB contamination (Mac & Edsall, 1991). Today, Lake Superior populations remain relatively stable and recruitment has been observed in lakes Huron (Riley et al., 2007), Michigan (Hanson et al., 2013), and Ontario (Lantry, 2015). Recent research suggests that domesticated strains used for reintroduction have variable fitness in contemporary Great Lakes environments (Larson et al., 2021; Scribner et al., 2018) and may be differentially contributing to recent recruitment. However, the biological mechanisms that underly these differences in fitness and recruitment remain unclear.

Genomic and transcriptomic approaches have been widely used to identify loci associated with adaptive diversity and ecotypic divergence in salmonids (Prince et al., 2017; Rougeux et al., 2019; Veale & Russello, 2017; Willoughby et al., 2018). This work has been partially driven by the publication of high‐quality genome assemblies and linkage maps for numerous salmonid species (Christensen, Leong, et al., 2018; Christensen, Rondeau, et al., 2018; De‐Kayne et al., 2020; Gagnaire et al., 2013; Lien et al., 2016; Pearse et al., 2019); however, genomic resources are notably lacking for Lake Trout. An annotated, chromosome‐anchored, genome assembly is arguably the most valuable resource for advancing genomic research on any species. A publicly available reference genome for Lake Trout would eliminate many challenges associated with conducting conservation‐oriented genetic research aimed at restoring ecotypic diversity and viable wild populations. Until recently, the assembly of non‐model eukaryotic genomes was prohibitively expensive, computationally challenging, and required the collaborative efforts of large genome consortia; however, the development of long‐read (“third generation”) sequencing technologies has to some extent eliminated these hurdles (Hotaling & Kelley, 2020; Whibley et al., 2020).

Long‐read sequencing data can be useful for scaffolding and filling gaps in existing, fragmented, short‐read assemblies (English et al., 2012). A number of assembly algorithms also seek to assemble contigs directly from long‐read sequencing data (Falcon; Chin et al., 2016; Canu; Koren et al., 2017; wtdbg2; Ruan & Li, 2020) and recent work suggests that this approach can be highly effective for assembling chromosome‐anchored salmonid genomes when combined with additional scaffolding information (De‐Kayne et al., 2020; also see RefSeq: GCF_002021735.2).

Salmonid genomes are highly complex and relatively difficult to assemble owing to the existence of large LORe regions (Robertson et al., 2017) and high repeat content (De‐Kayne et al., 2020; Kajitani et al., 2014; Lien et al., 2016). Sequencing low‐diversity individuals from inbred lines or homozygous individuals produced via chromosome set manipulations provides one route for simplifying the assembly process and correctly assembling regions with low levels of differentiation between homeologs. Previous salmonid genome assemblies have made use of doubled haploid individuals (Christensen, Leong, et al., 2018; Lien et al., 2016; Pearse et al., 2019) because these individuals are theoretically homozygous at all loci (but see Hansen et al., 2020). Additionally, long read sequencing data has been shown to be highly effective for assembling polyploid genomes (Du et al., 2020), and these data would probably improve our ability to resolve LORe regions in salmonids. For instance, De‐Kayne et al. (2020) recently published a highly contiguous assembly for European Whitefish (*Coregonus* sp. *balchen*); however, this assembly was produced using data from an outbred, wild‐caught, individual rather than a double haploid.

Here, we present a chromosome‐anchored reference genome for a female Lake Trout that was assembled using Pacific Bioscience long‐read sequencing data and scaffolded using a high‐density linkage map (Smith et al., 2020) and genome‐wide chromatin conformation capture followed by massively parallel sequencing (Hi‐C). We also produced a number of complementary resources including a custom repeat library and an interpolated recombination map in order to facilitate additional research on this important species. A publicly available set of gene annotations was also produced using the NCBI Eukaryotic Genome Annotation Pipeline. Additionally, we identify Lake Trout homeologs resulting from the salmonid specific autotetraploid event (Ss4R) and establish homologous relationships with chromosomes from other salmonid species.

## 2 MATERIALS AND METHODS

### 2.1 Crossing and sample collection

Gynogenetic double haploids were produced by fertilizing eggs with UV irradiated sperm, then pressure shocking embryos immediately following the first mitotic division (as described in Limborg et al., 2016; Thorgaard et al., 1983). Double haploid (DH) offspring were created at Iron River National Fish Hatchery using eggs and sperm collected from captive adult Lake Trout from the U.S. Seneca Lake brood stock. The U.S. Seneca Lake hatchery strain was entirely founded by early Autumn spawning Lake Trout initially collected from Seneca Lake, New York (see Page et al., 2003 and Krueger & Ihssen, 1995). Due to low survivorship of DH offspring (Komen & Thorgaard, 2007), we tested multiple UV and pressure shock treatments on eggs from five different females. Batches of 900 eggs from each female were fertilized with sperm that was irradiated for 140, 280, or 1260 s. Each batch was then split and subbatches were pressure shocked at 11,000 PSI for 5 min at either 6.5, 7, 7.5, 8, 8.5, 9, 9.5, or 10 h post‐fertilization. A total of 13,500 eggs were exposed to various UV and pressure shock treatments. One batch of 900 eggs from each female was also exposed to a control treatment which involved no sperm irradiation or pressure shock. Embryos were incubated in heath trays at ambient temperature until eye‐up stage (E^3^6 per Balon, 1980), with dead embryos being removed from trays on a daily basis. A single individual that survived past post‐embryo stage (*sensu* Marsden et al., 2021) was grown to a size of approximately 5 cm before being sampled post‐mortality and stored at –20°C. The post‐embryo stage in Lake Trout is characterized by a fully absorbed yolk sac, parr marks, and an inflated gas bladder (Marsden et al., 2021).

### 2.2 Laboratory methods

High molecular weight (HMW) DNA was extracted from white muscle sampled from the DH individual using a MagAttract HMW DNA Extraction kit (Qiagen). The manufacturer's recommended protocol was used except tissue digestion was done at room temperature for 140 min rather than 12–16 h at 55°C. Fragment size and yield were determined using pulse field gel electrophoresis and an AccuClear Ultra High Sensitivity DNA Quantification assay (Biotium). Prior to sequencing and assembly, we verified that the DH individual was completely homozygous at 15 microsatellite loci that are typically highly heterozygous in Lake Trout populations (Valiquette et al., 2014). A long‐read sequencing library was then prepared using the SMRTbell Template Prep Kit 1.0 (Pacific Biosciences), with the optional DNA Damage Repair step after size selection. Size selection was made for fragments >10 kb using a Blue Pippin instrument (Sage Science) according to the manufacturer recommended protocol for 20 kb template preparation. We used 5 µg of concentrated DNA as input for the library preparation reaction. Library quality and quantity were assessed using a genomic DNA Tape Station assay (Agilent), as well as Broad Range and High Sensitivity Qubit fluorometric assays (Thermo Fisher). Single‐Molecule Real Time sequencing was performed on the Pacific Biosciences Sequel instrument at the McGill Genome Centre (McGill University, Montreal, Canada, https://www.mcgillgenomecentre.ca/) using an on‐plate concentration ranging from 1.5–7.5 pM and the Sequel Sequencing Kit 2.0 with diffusion loading. 38 SMRTCells were run with 600 min movies and two SMRTCells were run with 1200‐min movies. All HMW DNA for the DH individual was expended over the course of PacBio sequencing runs. This necessitated the use of DNA from diploid individuals for generating additional libraries needed for scaffolding and polishing.

Hi‐C proximity ligation libraries were generated using tissue from a 7‐year‐old diploid female Lake Trout originating from the Killala Provincial hatchery strain. Four Hi‐C libraries were prepared using spleen and white muscle tissue using the Arima Hi‐C kit according to the manufacturer's protocol (A510008, Arima‐HiC_AnimalTissue_A160132_v00, Arima Genomics) and library preparation kits from Kapa Biosystems and Lucigen. Each Hi‐C library was spiked into a portion of an Illumina HiSeqX lane in order to assess how effectively reads could be mapped against the draft contig assembly. hicup version 0.7.2 (Wingett et al., 2015) within genpipes version 3.1.5 (Bourgey et al., 2019) was used to map Hi‐C sequencing reads against draft contigs. The Hi‐C library prepared using muscle tissue and prepared using the Arima‐Hi‐C and Lucigen Kits, was selected for further sequencing given that this library produced the highest proportion of reads mapped to draft contigs. This kit employs a restriction enzyme cocktail that digests chromatin at N^GATC and G^ANTC sequence motifs. The selected library was sequenced to high coverage in a single HiSeqX lane using the 2X150 bp paired end read format. Sequencing produced 182,781,953 paired end reads.

DNA was also extracted from fin tissue collected from an adult (diploid) female Lake Trout from the Seneca Lake broodstock using a MagAttract HMW DNA extraction kit (Qiagen) and protocol recommended by the manufacturer. Sequencing reads from this Seneca strain female were later used for contig polishing and correction (described below in Assembly and Scaffolding). The library was prepared using 100 ng of input DNA and the NEBNext Ultra Library Preparation Kit for Illumina (New England Biolabs). The library was sheared to approximately 400 bp using a Covaris M220 Ultrasonicator, amplified for eight cycles, and quantified using Quant‐It Picogreen dsDNA assays (Thermo Fisher) run in triplicate. Fragment size was assessed using a genomic DNA Tape Station assay (Agilent). The library was sequenced in multiplex with three other Lake Trout in two HiSeqX lanes using the paired end 2 × 150 read format. Sequencing produced 316,557,707 read pairs for this individual.

### 2.3 Assembly and scaffolding

Contig assembly using PacBio reads was carried out using the polished_falcon_fat assembly workflow run using the smrt analysis v3.0 pbsmrtpipe workflow engine provided with an installation of smrt link v5.0 (smrtlink‐release_6.0.0.47841; https://github.com/PacificBiosciences/pbsmrtpipe). Read metadata were extracted using the smrt analysis v3.0 data set tool with the merge option. Sequencing read metadata, pipeline settings, and an output directory were specified for the polished_falcon_fat pipeline option. Default assembly settings were used except genome size (HGAP_GenomeLength_str) was set to 3 gigabases (Gb), seed coverage (HGAP_SeedCoverage_str) was set to 40x, and the minimum read length to use a read as a seed (HGAP_SeedLengthCutoff_str) was set to 1000. Multiple settings were also changed. The resulting assembly settings file, read metadata file, and commands used to run the pipeline are available at https://github.com/smithsr90/LakeTroutGenome.

The polished_falcon_fat workflow uses FALCON assembly algorithm (Chin et al., 2013) and the quiver/arrow consensus tool (https://github.com/PacificBiosciences/GenomicConsensus) to generate a polished contig assembly. The Falcon method operates in two phases: First, overlapping sequence reads were compared to generate accurate consensus sequences with read N50 >10.9 Kb. Next, overlaps between the corrected longer reads were used to generate a string graph. The graph was reduced so that multiple edges formed by heterozygous structural variation were replaced to represent a single haplotype. Contigs were formed by using the sequences of nonbranching paths. Two supplemental graph cleanup operations were applied to improve assembly quality by removing spurious edges from the string graph: tip removal and chimeric duplication edge removal. Tip removal discards sequences with errors that prevent 5′ or 3′ overlaps. Chimeric duplication edges may be produced due to the production of chimeric molecules during library preparation or during the first sequence cleanup step and these errors artificially increase the copy number of a duplication. In a second and final workflow stage, the polished_falcon_fat workflow used the arrow consensus tool to perform error correction on the assembly using PacBio reads in order to generate an initial polished assembly. The resulting contigs were passed through a second round of error correction using Pilon in order to resolve SNP, indel, and local assembly errors before proceeding with scaffolding (https://github.com/broadinstitute/pilon ). The Illumina paired‐end sequencing data set from a Seneca strain female (described above) was mapped to draft contigs using BWA mem with default settings (Li, 2013). Reads with mapping qualities <20 were removed from the data set in order to exclude low quality alignments and reads mapping to multiple locations. Improperly paired reads were also excluded using samtools view (Li et al., 2009). The resulting filtered bam file was used as input for Pilon with the ‐‐fix all ‐‐mindepth 5, and ‐‐diploid options. Pilon was run prior to scaffolding in order to identify and correct local assembly errors that could potentially cause downstream scaffolding errors.

We adopted a multifaceted scaffolding approach leveraging information from Hi‐C sequencing and a high‐density linkage map for Lake Trout (Smith et al., 2020). Hi‐C reads were mapped to Pilon corrected contigs with default setting using the Arima Genomics Mapping pipeline (Arima Genomics, https://github.com/ArimaGenomics/mapping_pipeline), which included four primary steps. First, forward and reverse reads were mapped to the reference genome using bwa version 0.7.17 (Li, 2013) separately. Next, the 5’ end of the mapped reads were trimmed. samtools version 1.9 (Li et al., 2009) was then used to filter reads with mapping qualities <10 in order to remove low quality alignments and reads mapping to multiple locations. Finally, picard version 2.17.3 (https://broadinstitute.github.io/picard/) was used to add read group information and mark duplicate reads. The resulting BAM file was used as input for salsa v2.2 (Ghurye et al., 2017) run with default settings (three iterations). We also tested Salsa2 using five iterations and compared results with those produced using default settings by calculating Spearman's rank order correlation coefficients between the order of loci on the Lake Trout linkage map (Smith et al., 2020) and the order of loci on the 50 largest scaffolds. Linkage mapped RAD contigs were aligned to the reference assembly using Minimap2 (Li, 2018) using the ‐asm5 option. RAD contigs with mapping qualities less than 60 were removed before calculating correlation coefficients using the r function cor from the stats package (R Core Team, 2017) and the method argument set to “spearman.”

Additional scaffolding was carried out using chromonomer v1.13 (Catchen et al., 2020). The assembly was initially scaffolded using default settings, which yielded chromosome length scaffolds with a high degree of concordance with the linkage map; however, structural differences between the linkage map and scaffolds were apparent on six chromosomes. In order to resolve these inconsistences, we aligned the full set of PacBio subreads to the assembly using minimap2 (Li, 2018) using the preset option for PacBio data. The resulting bam file was sorted, indexed, and per‐base coverage was calculated for all positions using samtools depth with the ‐‐aa option. We then ran a second round of Chromonomer using the ‐‐rescaffold, ‐‐depth, and depth_stdevs = 2 options, which allowed for gaps to be opened in contigs if the site‐specific depth within a sliding window of 1000 base pairs was >2 standard deviations from the mean, suggesting an assembly error. This resulted in an assembly with improved concordance with the linkage map; however, linkage group 41 still exhibited a large inversion relative to the scaffolds. We determined the approximate location of this assembly error by identifying the pair of linkage mapped loci for which the level of discordance between the linkage map and assembly was maximized. The scaffold was manually broken and reoriented using an existing gap that existed between these two loci.

Gaps were filled using pbjelly from pbsuite v15.8.24 (English et al., 2012). All PacBio reads were aligned to the draft assembly using Minimap2 using the ‐pb preset option and reads mapping within 5000 base pairs of a gap were retained for gap filling using bedtools intersect (Quinlan & Hall, 2010). Retained reads were re‐mapped with blasr v5.3.2 (Chaisson & Tesler, 2012) using the options ‐‐minMatch 11, ‐‐minPctIdentity 75, ‐‐bestn 1, ‐‐nCandidates 10, ‐‐maxScore ‐‐500, and ‐‐fastSDP. The “maxWiggle” argument was set to 100 kilobases (Kb) for the PBJelly assembly stage in order to account for gaps of unknown length. After filling gaps, we corrected single nucleotide and short indel errors by running 3 iterations of Polca (distributed with masurca v. 3.4.2; Zimin & Salzberg, 2020) using Illumina data from a Seneca strain female as input. Polca was chosen because this error correction approach has been shown to be more effective for correcting single nucleotide and indel errors than comparable tools (Zimin & Salzberg, 2020). Default settings were used except low quality alignments (MQ < 10) and alignments overlapping gaps were removed from bam files using bedtools intersect (Quinlan & Hall, 2010) prior to running the Polca variant calling step.

Illumina paired end data from the same individual used for genome polishing and PacBio data from one SMRTcell were aligned to the Arctic Char (*Salvelinus alpinus*) mitochondrial genome (RefSeq: NC_000861.1) in order to obtain reads useful for assembling the Lake Trout mitochondrial genome. Reads were aligned using Minimap2 using the sr and map‐pb present options for short‐reads and long‐reads, respectively. Reads aligning to the Arctic Char mitochondrial genome were extracted from original fastq files using seqtk subseq (https://github.com/lh3/seqtk) and hybrid assembly was conducted using unicycler v0.4.8 (Wick et al., 2017) using the settings ‐‐min_fasta_length 15000 and ‐‐keep 0. Unicycler implements a hybrid‐assembly approach using Spades (Bankevich et al., 2012), SeqAn (Döring et al., 2008), and Pilon. First, spades (v3.13.1) was used to assemble Illumina short‐reads and contigs with graph coverage less than half the median coverage were removed due to potential contamination from the nuclear genome. Contigs were then scaffolded using long‐reads and SeqAn (Döring et al., 2008) was used to generate gap consensus sequences. Finally, Pilon was used to resolve assembly errors using short‐read alignments as input. The resulting mitochondrial genome assembly was aligned to all salmonid sequences in the NCBI nucleotide collection using blastn and standard settings in order to verify that it was consistent with previous Lake Trout mitochondrial assemblies. A neighbour‐joining tree constructed from blast pairwise alignments was exported from the NCBI website and is available in Figure S6.

### 2.4 Assembly quality control

We used multiple approaches to assess the accuracy, contiguity, and completeness of the genome assembly. First, we determined the proportion of the genome that was recovered in our assembly by comparing total assembly size with an estimate of genome size based on the distribution of k‐mer frequencies from Illumina paired‐end 2 × 150 data generated using DNA from a Seneca strain female. The frequency of all 19 mers in the read data was calculated using the count function in jellyfish v2.2.6 (Marçais & Kingsford, 2011) with the options ‐m 19 and ‐C. K‐mer counts were then exported to the histogram format using the histo function. This file was used as input for genomescope v1.0 (http://qb.cshl.edu/genomescope/; Vurture et al., 2017) with read length set to 150 bp and k‐mer length set to 19.

Basic assembly statistics were calculated using the program summarizeassembly.py from pbsuite v15.8.24 (English et al., 2012). Statistics included total assembly size, contig and scaffold N50s, and minimum and maximum contig and scaffold lengths. Assembly statistics were calculated with and without gaps. Contig and scaffold N50s and counts were obtained for 14 additional salmonid assemblies from NCBI for comparison. Single base consensus accuracy was estimated during each iteration of polishing with Polca as the proportion of bases in input sequences overlapping detected errors.

Next, we calculated percentages of complete singleton, complete duplicated, fragmented, and missing Benchmarking Single‐Copy Orthologues (BUSCOs) for seven chromosome‐level salmonid assemblies and compared these with scores for the Lake Trout assembly discussed here. These included genomes for Brown Trout (*Salmo trutta*; GCA_901001165.1), European Whitefish (*Coregonus sp*. *balchen*; GCA_902810595.1; De‐Kayne et al., 2020), Atlantic Salmon (*Salmo salar*; GCA_000233375.4; Lien et al., 2016), Coho Salmon (*Oncorhynchus kisutch*; GCA_002021735.1), Rainbow Trout (*Oncorhynchus mykiss*; GCA_002163505.1; Pearse et al., 2019), Chinook Salmon (*Oncorhynchus tshawytscha*; GCA_002872995.1; Christensen, Leong, et al., 2018), and Dolly Varden (*Salvelinus malma*; GCA_002910315.1; Christensen, Rondeau, et al., 2018). It should be noted that the assembly originally produced for Arctic Char (GCA_002910315.1; Christensen, Rondeau, et al., 2018, referred to as the Dolly Varden assembly here) was later found to be from a Dolly Varden or potentially a Dolly Varden – Arctic Char hybrid (see Shedko, 2019 and Christensen et al., 2021). BUSCO scores were also calculated for the Northern Pike genome (*Esox lucius*; GCA_000721915.3; Rondeau et al., 2014), a member of the order *Esociformes* that is commonly used as a pre‐Ss4R outgroup species. BUSCO scores were calculated using busco v4.0.6, the actinopterygii_odb10 database (created 20 November 2019), and the ‐genome option.

Finally, we aligned the linkage mapped contigs from Smith et al. (2020) to the final assembly and calculated Spearman's rank order correlation coefficients between physical mapping locations and the order of loci along linkage groups. Linkage mapped contigs were aligned to the reference assembly using Minimap2 using the ‐asm5 preset parameters and the resulting sam file was filtered to exclude contigs with mapping qualities less than 60. Correlation coefficients were calculated using the cor function in r (R Core Team, 2017) with the method argument set to “spearman.” Correlation coefficients were then converted to absolute values using the abs function in order to compare chromosomes and linkage groups with reversed orientations.

### 2.5 Repetitive DNA

**FIGURE 2:**
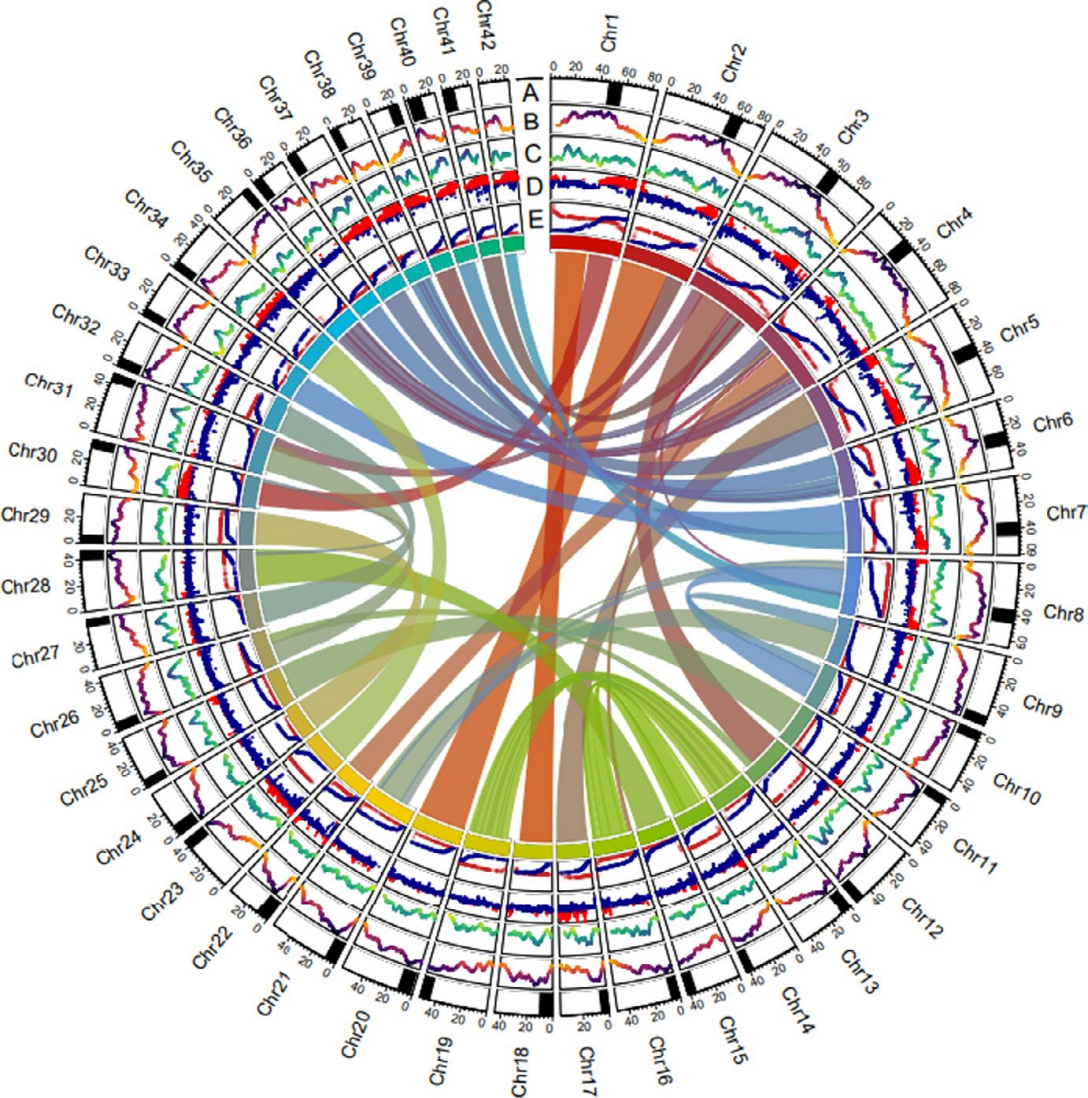
Circos plot displaying centromere positions, Tcl‐Mariner abundance, density of annotated protein coding genes, local homeolog sequence identity, male and female Lake Trout (*Salvelinus namaycush*) linkage maps, and homeolog pairs resulting from Ss4R. (A) Black boxes in the outside ring display the mean mapping positions (±5 Mb) for centromere associated RAD loci from Smith et al. (2020). (B) The second ring displays Z‐transformed Tcl‐Mariner repeat abundance in 5 Mb sliding windows with an offset of 100 kilobases. (C) The third ring displays the density of annotated genes in 5 Mb sliding windows with an offset of 100 kilobases. (D) The fourth ring displays local homeolog identity between syntenic blocks detected by SynMap2. Red points correspond to windows with elevated sequence identity putatively resulting from delayed rediploidization (posterior probability >0.5). Blue points correspond to windows with elevated sequence divergence between homeologs. (E) The fifth ring displays map distance (centimorgans) for male (red) and female (blue) linkage maps (*y*‐axis) versus physical distance (*x*‐axis) for each of the 42 chromosomes. Connections are drawn between syntenic blocks identified by symap v5 putatively resulting from Ss4R

A custom repeat library was created using repeatmodeler v2.0.1 (Flynn et al., 2020) and repeats were subsequently classified using repeatclassifier (Smit et al., 2015). Repeats were then masked using repeatmasker (Smit et al., 2015) and the output of repeatmasker was used to determine the genome‐wide abundance of different repeat families and the relative density of repeat types across chromosomes. The density of the most abundant repeat type (Tcl‐mariner) was visualized across chromosomes using the r‐package circlize (Gu et al., 2014; Figure 2).

### 2.6 Homeolog identification and synteny

We performed a self‐versus‐self synteny analysis using symap v5 (Soderlund et al., 2006, 2011) to identify Lake Trout homeologs resulting from the salmonid specific autotetraploid event (Lien et al., 2016; Macqueen & Johnston, 2014). Prior to running symap, we hard‐masked the genome using repeatmasker v4.1.0 (Smit et al., 2015) using our custom repeat library as input and RMblast as the search engine (‐e ncbi). Nucmer (Marçais et al., 2018) was used for SyMap alignments and options were set to min‐dots = 30, top_*n* = 2, and merge_blocks = 1. We then used Symap to identify blocks of synteny between Lake Trout and Dolly Varden, Rainbow Trout, and Atlantic Salmon. These alignments were conducted using Promer (Marçais et al., 2018), and we used the options min_dots = 30, top_*n* = 1, merge_blocks = 1, and no_overlapping_blocks = 1. Results from self‐versus‐self synteny analysis were visualized using the r package circlize (Gu et al., 2014). Additionally, we identified syntenic relationships with Northern Pike using synmap2 (Haug‐Baltzell et al., 2017). We used the last algorithm to align genomes, DAGChainer to identify syntenic blocks (‐D20, ‐A5), Quota Align Merge to merge syntenic blocks (‐Dm 0), and Quota Align (Overlap Distance = 40) to enforce a 1‐to‐2 ploidy relationship between Northern Pike and Lake Trout (Haas et al., 2004; Tang et al., 2011).

We also repeated our self‐versus‐self synteny analysis using SynMap2 while enforcing 2‐to‐2 synteny relationships. Sequence identity between homeologs was extracted from the output of SynMap2 for all merged regions composed of more than 1000 blocks. We then computed the moving average of local homeolog identity across chromosomes using sliding windows containing 200 blocks. We then fit a Gaussian mixture model to the distribution of homeolog identities using mixtools (Benaglia et al., 2009) and the function normalmixEM (*k* = 2) after observing that the values were bimodally distributed. Posterior probabilities of assignment to clusters with high and low homeolog divergence were determined for each window in addition to cluster means and mixing proportions (lambdas) for the data set. Results from the Lake Trout‐versus‐ other salmonids synteny analysis were visualized using the Chromosome Explorer option in symap v5. Syntenic relationships between Lake Trout and Northern Pike were visualized as a dotplot generated in r (Figure S5).

### 2.7 RNA sequencing and gene annotation

RNA samples derived from liver were obtained from the offspring of Seneca Lake hatchery strain fish held within the Ontario Ministry of Natural Resources and Forestry (OMNRF) hatchery system. Offspring were produced using four males and four females in a full factorial mating cross, by dry‐spawning anaesthetized fish (anesthetic: 0.1 g/L MS‐222; Aqua Life, Syndel Laboratories Ltd.). Eggs (140 ml) were stripped from each female, divided evenly among four jars, and fertilized by pipetting milt directly onto them. After fertilization, embryos were transported to the Codrington Fish Research Facility (Codrington, Ontario, Canada) where they were transferred from the jars into perforated steel boxes with one family per box. These boxes were contained in flow‐through tanks receiving freshwater at ambient temperature (5–6°C) and natural photoperiod under dim light. When the embryos fully absorbed their yolk sacs and were ready to feed exogenously (i.e., free embryos; approximately March 2016), 14 individuals from each family were randomly selected and split into two groups of seven, then transferred into one of four larger (200 L) tanks.

Tissue sample collection occurred between 28 June to 9 August 2016. The mean body mass and fork length of fish at sampling was 3.60 g (SD = 1.30) and 7.23 cm (SD = 0.78), respectively. Each fish was euthanized in a bath of 0.3 g/L of MS‐222 and dissected to remove the whole liver. The liver was gently blotted on a laboratory wipe and stored in RNAlater (Invitrogen, Thermo Fisher Scientific) for 24–48 h at room temperature. RNALater was pipetted from the liver tissue and the samples were stored at –80°C until RNA isolation. Liver tissues were homogenized individually in 2 ml Lysing Matrix D tubes (MP Biomedicals) with 1 ml of Trizol reagent (Invitrogen, Thermo Fisher Scientific). RNA was extracted from the homogenate using phenol‐chloroform extraction (Chomczynski & Sacchi, 2006). RNA was precipitated with RNA precipitation solution (Sambrook & Russell, 2001) and isopropanol, and washed with 75% ethanol. RNA samples were resuspended in nuclease‐free water (Thermo Fisher Scientific). The purity and concentration of the RNA were initially determined using a NanoDrop‐8000 spectrophotometer. RNA quality was also assessed using a Bioanalyzer (Agilent) and resulting RNA integrity numbers (RIN). All RNA samples met our minimum RIN threshold of 7.5.

RNA sequencing was performed over 2 years. Twenty‐four samples were sent to The Centre for Applied Genomics (Sick Kids Hospital, Toronto, Ontario, Canada) in 2018, and another 30 samples were sent to the Centre d'expertise et de services Génome Québec (Montreal, Quebec, Canada; https://cesgq.com/) in 2020. cDNA libraries were produced by enriching the poly(A) tails of mRNA with oligo dT‐beads using the NEBNext Ultra II Directional polyA mRNA Library Prep kit (New England Biolabs). The group of 24 individuals was sequenced in 2.5 Illumina HiSeq 2500 lanes using 2 × 126 bp paired end reads. The additional 30 individuals were sequenced in three Illumina HiSeq 4000 lanes using 2 × 126 bp paired end reads. Data were deposited in sequence read archives associated with BioProject PRJNA682236. These sequencing reads, along with those from two previous RNAseq experiments for liver and muscle tissue (Goetz et al., 2010; Goetz et al., 2016: SRA Accessions SRS005644 and SRS387865), were used as input for NCBI’s Eukaryotic Genome Annotation Pipeline (Thibaud‐Nissen et al., 2016). A collection of 3547 Atlantic Salmon transcripts were also used as input. The annotation was produced by NCBI during January, 2021. For a complete record of data sources used for transcript alignments and gene prediction see: https://www.ncbi.nlm.nih.gov/genome/annotation_euk/Salvelinus_namaycush/100/).

### 2.8 Recombination rates and centromeres

Sex averaged recombination rates were estimated across chromosomes using the sliding window interpolation approach implemented in mareymap (Rezvoy et al., 2007). Restriction site associated DNA (RAD) contigs from the Lake Trout linkage map (Smith et al., 2020) were mapped to chromosomes using minimap2 using the ‐asm5 preset option and reads with mapping qualities <60 were removed. At this point, RAD loci overlapping centromere mapping intervals for each linkage group were extracted and the centromere centre was considered to be the mean mapping position for centromere associated RAD tags. Centromere positions were visualized using the r‐package circlize (Gu et al., 2014).

In order to remove contigs with anomalous mapping positions that could bias recombination rate estimates, we fit a loess model describing linkage map position as a function of physical position for each chromosome, extracted model residuals, and removed markers with residuals that were greater than one standard deviation from the mean. Loess models were fit using the loess function in r with the span argument set to 0.2 and the degree argument set to 2. The remaining markers were output to MareyMap format and were manually curated using mareymap Online (Siberchicot et al., 2017). A sex averaged recombination map was calculated using sliding window interpolation and exported from the program (Appendix S1 – Recombination Map).

## 3 RESULTS

### 3.1 Sequencing, assembly and scaffolding

Of 13,500 embryos exposed to UV irradiation and pressure shock treatments, only two individuals survived beyond post‐embryo stage and one individual survived to a size of approximately 5 cm. This individual was found to be homozygous at all 15 genotyped microsatellite loci, suggesting that chromosome set manipulations were successful at inducing double haploidy. HMW DNA extraction yielded a DNA concentration of 70 ng/µl based on nanodrop readings. We proceeded with PacBio sequencing, and produced a data set with an estimated genome coverage of 89x, with 53x coverage provided by reads longer than 12 Kb in length.

The Falcon‐based assembly pipeline and polishing with Arrow and Pilon yielded an initial assembly with 8321 contigs, a total length of 2.3 Gb, and a contig N50 of 1.3 megabases (Mb) with a maximum contig length of 19.6 Mb (Table 1). Our analysis comparing the correlation between the Lake Trout linkage map and Hi‐C scaffolds indicated that three iterations of Salsa (the default setting) produced moderately large scaffolds suitable for downstream use. We opted to use these settings for scaffolding. Salsa v2.2 split multiple contigs, resulting in 8367 contigs with an N50 of 1.25 Mb and 5171 scaffolds with an N50 of 5.15 Mb. Additional scaffolding with Chromonomer v1.13 increased scaffold N50 to 44 Mb and reduced the total number of scaffolds to 4122. Chromonomer v1.13 also reduced contig N50 to a small degree due to the insertion of additional gaps at likely misassembles. Scaffolding with Hi‐C and the Lake Trout linkage map ultimately allowed us to assign 84.7% of the genome to chromosomes. Gap filling with PBJelly increased scaffold N50 to 44.97 Mb, increased the total assembly size to 2.345 Gb, and increased contig N50 to 1.8 Mb (Table 1). Gap filling increased the maximum contig length to 34.78 Mb and the maximum scaffold length to 98.19 Mb. The consensus accuracy reported during the third round of error correction with Polca was 99.9959%. The polished assembly was submitted to GenBank for public use (accession GCA_016432855.1).

**TABLE 1:** General summary statistics for the Lake Trout (*Salvelinus namaycush*) genome assembly. The total number of chromosomes, scaffolds (including chromosomes), and contigs are listed in the top row. Metrics reported for chromosomes and scaffolds include gaps of unknown length. Consensus accuracy was obtained from the output of POLCA after running three iterations of the program

	Chromosomes	Scaffolds	Contigs	Gaps
Count	42	4120	7378	3258
Minimum length (bp)	22,041,605	9606	84	100
Mean length (bp)	47,175,710	569,295	317,859	100
Max length (bp)	98,200,354	98,200,354	34,788,501	100
Total length (bp)	1,981,379,816	2,345,496,355	2,345,170,555	325,800
N50 (bp)	48,336,861	44,976,251	1,804,090	100
N90 (bp)	34,530,387	249,999	114,532	100
N95 (bp)	26,015,404	84,453	61,568	100
Consensus accuracy (%)	‐	‐	99.9959	‐

### 3.2 Assembly quality control

We estimated the total haploid genome size for Lake Trout to be between 2.119 and 2.122 Gb using k‐mer analysis and GenomeScope v1.0, with 38% of the genome composed of unique sequence and 62% composed of repetitive sequence (Table S1, Figure S1). Heterozygosity for the sample used for polishing was estimated to be between 2.78 and 2.9 heterozygous sites per 1000 base pairs. It should be noted that the individual used for polishing was a diploid and not a gynogenetic double haploid. The estimated coverage for the sample used for genome‐size estimation was 16×, which should be sufficient for k‐mer based methods (Williams et al., 2013).

**FIGURE 3:**
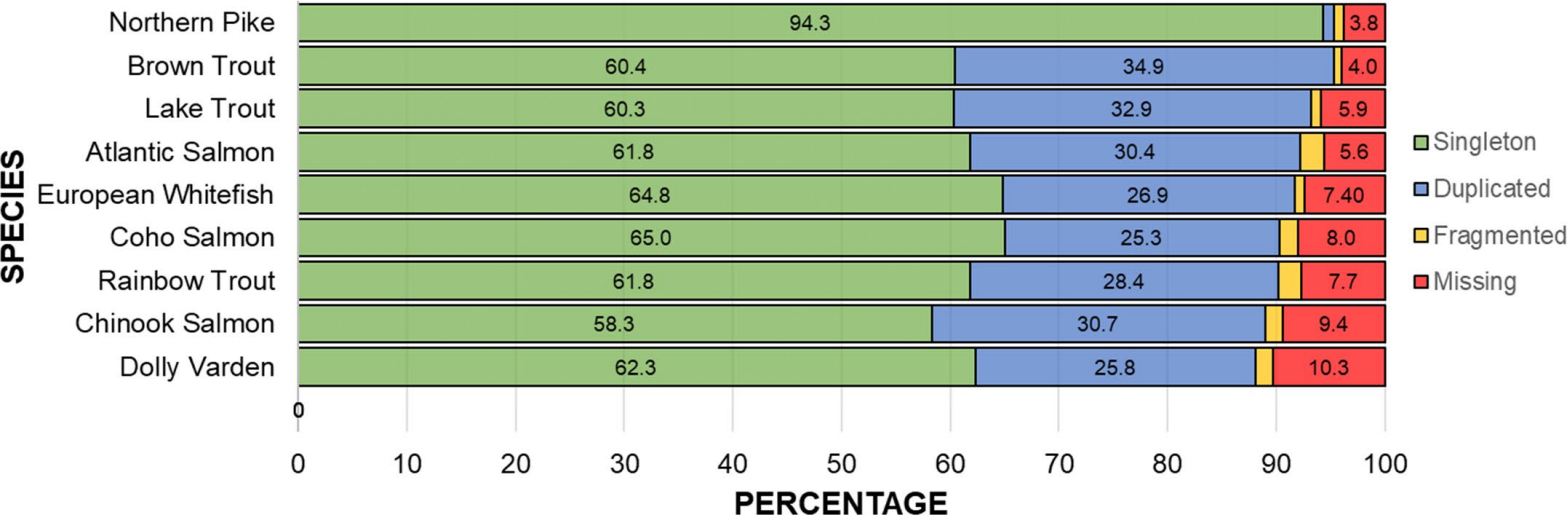
Comparison of BUSCO scores across multiple chromosome‐level salmonid assemblies. Scores for the preduplication outgroup species (Northern Pike; *Esox lucius*) are also included for comparison. Assemblies are listed top to bottom according to the total percentage of complete BUSCOs. Complete single‐copy, complete duplicated, fragmented, and missing BUSCO percentages are delineated with green, blue, yellow, and red bars, respectively

We recovered 93.2% of BUSCO genes with 60.3% and 32.9% being present as singletons and duplicates, respectively (Figure 3; Table S2). The salmonid genomes evaluated recovered between 88.1% and 95.3% complete BUSCOs with between 25.3% and 34.9% being duplicated and between 58.3% and 65% being singletons. The proportion of duplicated BUSCOs in the Lake Trout genome was the second highest among the compared salmonid genomes (32.9%) and appears to be comparable to the Brown Trout genome (GCA_901001165.1; River Trout), which was also assembled using Falcon (Falcon‐unzip) and polished using a method based on the Freebayes variant caller (Garrison & Marth, 2012).

We found that the mitochondrial genome assembly produced here falls within a monophyletic group entirely composed of mitochondrial sequences previously generated for Lake Trout (Figure S6; Schroeter et al., 2020). The assembly was most similar to one produced for a Lake Trout sampled from Lake Ontario, Pennsylvania (99.96% Identity; Accession: MF621746.1). The Seneca Lake hatchery strain is heavily stocked in Lake Ontario and appears to have elevated fitness in this environment (Perkins et al., 1995).

The mean linkage map versus Hi‐C scaffold Spearman's correlation was 0.89 across the 50 largest Hi‐C scaffolds. These were calculated prior to integrating linkage information from the map. Thirty‐three of the 50 largest Hi‐C scaffolds had correlations >0.95 and 42 had correlations >0.8. Spearman's rank order correlations between finished chromosomes and the linkage map ranged from 0.89 to 1.0 for the 42 Lake Trout chromosomes. High correlation coefficients are expected in this case because the linkage map was used to scaffold chromosomes. The mean correlation coefficient was 0.98 and 39 of 42 finished chromosomes had correlations greater than or equal to 0.96, suggesting that the linkage map and genome assembly provide a concordant representation of the order of loci along chromosomes (Figure 2e).

### 3.3 Repetitive DNA

RepeatModeler 2 identified 2810 interspersed repeats and 462 of these were classified by RepeatClassifier. RepeatMasker reported that 53.8% of the Lake Trout genome is composed of sequences from this repeat library. A total of 13.04% of the genome was composed of retroelements, with 10.47% being LINEs and 2.57% being LTR elements, and 9.97% of the genome was composed of DNA transposons. As has been observed in other salmonids, TcMar‐Tc1 was the most abundant superfamily and these repeats were most abundant near centromeres (Figure 2; Lien et al., 2016; Pearse et al., 2019). A total of 30.79% of the genome was composed of interspersed repeats that were not classified by RepeatClassifier (Table 2).

**TABLE 2:** Number of elements, total sequence length, and percent of the Lake Trout (*Salvelinus namaycush*) genome occupied by retroelements, DNA transposons, and other repeat types

	No. elements	Length	Percent
Retroelements	551,376	305,755,720	13.04
SINEs	0	0	0.00
Penelope	11,724	3,138,292	0.13
LINEs	483,866	245,479,169	10.47
CRE/SLACS	0	0	0.00
L2/CR1/Rex	337,340	178,461,635	7.61
R1/LOA/Jockey	9131	2,778,587	0.12
R2/R4/NeSL	705	573,357	0.02
RTE/Bov‐B	28,238	14,293,769	0.61
L1/CIN4	12,257	6,142,123	0.26
LTR Elements	67,510	60,276,551	2.57
BEL/Pao	1533	1,173,630	0.05
Ty1/Copia	1427	1,007,823	0.04
Gypsy/DIRS1	55,237	49,788,865	2.12
Retroviral	9313	8,306,233	0.35
DNA transposons	533,707	233,872,078	9.97
hobo‐Activator	34,814	15,807,935	0.67
Tc1‐IS630‐Pogo	473,487	209,441,783	8.93
En‐Spm	0	0	0.00
MuDR‐IS905	0	0	0.00
PiggyBac	9091	3,370,797	0.14
Tourist/Harbinger	3105	834,759	0.04
Other (Mirage, P‐elements, Transib)	1104	292,535	0.01
Rolling‐circles	348	227,654	0.01
Unclassified:	2,885,512	722,299,456	30.79
All interspersed repeats		1,261,927,254	53.80

### 3.4 Homeolog identification and synteny

Self‐versus‐self synteny analysis conducted using symap v5 identified 126 syntenic blocks shared between putative Lake Trout homeologs (Figure 2). Blocks ranged in size from 477,153 bp to 57,126,662 bp. Fifty‐two blocks were longer than 10 Mb and 70 were longer than 5 Mb (Figure 2, inner links). The distribution of local homeolog identity was bimodal and our Gaussian mixture model estimated that the means of these two distributions were 82.72% and 90.64%. The lambda estimates for the model were 0.5848 and 0.4152 suggesting that approximately 41.52% of the Lake Trout genome exhibits a signal of delayed rediploidization (Figure 2d). Loci with elevated homeolog sequence identity were primarily located near the telomeres of metacentric chromosomes (Chr1‐Chr8); however, one pair of acrocentric homeologs (Chr23 and Chr34) also exhibited elevated sequence identity.

We identified 50 syntenic blocks shared between Rainbow Trout and Lake Trout and identified homologous Rainbow Trout chromosomes for all Lake Trout chromosomes. Syntenic blocks shared between these two species ranged in size from 1.9 Mb to 97.2 Mb. Symap identified homologous chromosomes in Atlantic Salmon for all chromosomes except Lake Trout chromosomes 32 and 39. However, we expect that Lake Trout chromosome 39 is homologous to a region of Atlantic Salmon chromosome 2 and Lake Trout chromosome 32 is homologous with a region of Atlantic Salmon chromosome 14 based on the size of missing synteny blocks. Specifically, Lake Trout chromosomes 32 and 39 are 37.24 Mb and 23.59 Mb in length, respectively. The two regions with missing homology in Atlantic Salmon on chromosomes 2 and 14 are approximately 27.9 Mb and 42.9 Mb, respectively. Fifty‐four syntenic blocks were detected between these species that ranged in size from 208,516 bp to 88 Mb. We identified 42 syntenic blocks shared between Dolly Varden and Lake Trout and identified homologues for all chromosomes except Chr 41. Syntenic blocks ranged in size from 6.8 Mb to 79.9 Mb. Pre‐Ss4R ancestral chromosomes were also detected in Northern Pike (Figures S2–S5).

### 3.5 Genome annotation

We generated a total of 3.45 billion RNA‐seq reads from liver tissue that were subsequently used as input for the NCBI Eukaryotic Genome Annotation Pipeline v8.5 (9 July 2020 release date). An additional 528,760 reads were used from previous Lake Trout gene expression studies. A total of 86% of reads were aligned to the genome assembly, and 12 Lake Trout transcripts from GenBank and 3547 known Atlantic Salmon transcripts from RefSeq were ultimately used as input for the pipeline.

The pipeline produced annotations for 49,668 genes and pseudogenes. A total of 3307 nontranscribed pseudogenes and two transcribed pseudogenes were identified. Gene length ranged from 53 to 1,198,409 bp, with a median length of 8676 bp. Gene densities for chromosomes ranged from 15.45 to 31.39 genes/Mb with an average genome‐wide density of 21.07 genes/Mb (Figure 2c). A total of 422,014 exons were identified, with between 1 and 224 exons per transcript (mean = 10.31, median = 8).

### 3.6 Recombination rates and centromeres

We were able to map between 1 and 238 centromere‐associated RAD contigs per chromosome and determine approximate centromere locations for all chromosomes except chromosome 42 (Table S4; Figure 2a). Smith et al. (2020) did not determine the location of the centromere for chromosome 42, which prohibited us from identifying its location here. Across all chromosomes, we mapped 35 centromere‐associated RAD loci per chromosome on average. Between 39 and 238 centromeric loci were mapped to metacentric chromosomes (mean = 93), while between 1 and 59 loci were mapped for acrocentric or telocentric chromosomes (mean = 21).

In all, 14,438 linkage‐mapped contigs were mapped to the genome with mapping qualities greater than 60 (Figure 2e). A total of 11,232 loci were retained for recombination rate estimation after manual curation and filtering using loess model residuals. We determined the mean sex averaged recombination rate to be 1.09 centimorgans/Mb, with recombination rates varying between 0 and 6.58 centimorgans/Mb across the genome. The interpolated recombination map produced by MareyMap is available in Appendix S1 – Recombination Map.

## 4 DISCUSSION

The adoption of multiple complementary scaffolding approaches resulted in an assembly of similar quality to the best available salmonid genomes. Multiple lines of evidence suggest that the genome presented here represents a nearly complete and accurate model of the female Lake Trout genome. First, the total size of the finished genome was slightly greater than the genome size estimate obtained from GenomeScope (2.3 Gb vs. 2.1 Gb). Pflug et al. (2020) found that k‐mer based methods for genome size estimation tend to underestimate genome size by 4.5% on average, so this result is not entirely unexpected. Additionally, BUSCO scores were similar to those obtained for the highest quality salmonid genomes available at the time of analysis. Among the genomes examined, Brown Trout, Lake Trout, Atlantic Salmon, and European Whitefish had the highest proportion of complete BUSCOs (95.3%, 93.2%, 92.2%, and 91.7%, respectively). Overall, Lake Trout BUSCO scores were most similar to those obtained for Brown Trout; however, the proportion of missing BUSCOs was 1.9% higher for Lake Trout and the proportion of complete duplicated BUSCOs was 2% lower suggesting that some duplicated regions might be missing from the Lake Trout genome. Nonetheless, these two assemblies had the highest percentage of complete BUSCOs and the highest percentage of complete duplicated BUSCOs out of the genome assemblies examined, suggesting that these two assemblies more effectively resolve LORe regions with high sequence similarity. Furthermore, the order of loci on the Lake Trout linkage map and the order of loci on Lake Trout chromosomes was shown to be highly concordant; however, it should be noted that the linkage map cannot be considered an independent source of validation. The genome presented here is also highly contiguous, with a contig N50 higher than any published salmonid genome at the time of analysis (but see the recently released assemblies for Arlee Strain Rainbow Trout ‐ GCF_013265735.2 and Atlantic Salmon ‐ GCA_905237065.2).

Interestingly, the PacBio data used for assembly were of similar coverage to the data used for assembling the European Whitefish genome (De‐Kayne et al., 2020); however, the Lake Trout genome contig N50 is >3x higher (1.8 Mb vs. 0.53 Mb; Table S3). It is worth noting that this assembly was produced using a different assembler (wtdbg2; Ruan & Li, 2020); however, an assembly with a contig N50 of 211 Kb was also generated from these data using Falcon (De‐Kayne et al., 2020). There are at least two reasonable explanations for the pronounced difference in contig N50 between the Lake Trout genome and European Whitefish assemblies produced using Falcon and wtdbg2. First, the European Whitefish genome was assembled using DNA from a wild‐caught, outbred individual rather than a double haploid. Second, the European Whitefish genome was not gap filled after scaffolding. Gap filling the Lake Trout genome with PBJelly increased contig N50 by 561,496 bp, which partially explains the difference.

Additionally, our analysis of homeolog sequence identity across the Lake Trout genome indicates that regions exhibiting delayed rediploidization (i.e., LORe regions; Robertson et al., 2017) are primarily associated with metacentric chromosomes and their acrocentric homeologs in Lake Trout. We identified one pair of acrocentric homeologs with elevated sequence identity (Chr23 and Chr34). Similar to Lien et al. (2016), these results suggest that homeologous pairing might not necessarily require one chromosome to be metacentric as suggested by Kodama et al. (2014). Interestingly, the region with elevated homeolog sequence identity on chromosome 4 does not appear to be associated with one of the telomeres. A previous quantitative trait locus mapping study suggested that this chromosome harbours the sex determining gene (*SdY*) in Lake Trout (Smith et al., 2020).

It is important to note that the assembly presented here does not represent a true haploid assembly even though contigs were assembled using DNA from a double haploid individual. The assembly was error corrected using sequencing reads from a diploid female, the linkage map was generated using families from multiple hatchery strains (Smith et al., 2020), and Hi‐C data were generated from a diploid individual from a separate strain. Therefore, the chromosome sequences presented here represent consensus sequences for female Lake Trout (from the Seneca L. strain) rather than haplotypes existing within the DH individual we sequenced. Additionally, PacBio assemblies are known to have an elevated prevalence of short indel errors relative to short read assemblies. These errors can interfere with annotation and necessitate error correction using short‐read data (Watson & Warr, 2019). For this assembly, we excluded multimapping reads with low mapping qualities in order to avoid homogenizing variation between homeologs during error correction. This could have resulted in an elevated prevalence of short indel errors within duplicated regions with high homeolog sequence identity, which could make it more difficult to annotate genes in these regions.

Nonetheless, the genome presented here represents a significant improvement compared to existing genomic resources for the genus *Salvelinus* (Figure 3, Tables S2 and S3). Improvements could probably be made to the assembly using supplementary scaffolding resources such as a higher density linkage map or optical map (Pan et al., 2020). The annotation could also be improved by generating additional RNA‐seq data. Nevertheless, the number of annotated genes and pseudogenes (*n* = 49,668) is similar to what has been obtained for other recent salmonid assemblies (e.g., Chum salmon, *Oncorhynchus keta*, GCF_012931545.1, *n* = 45,643; Sockeye salmon, *Oncorhynchus nerka*, GCF_006149115.1, *n* = 46,184; Dolly Varden, *Salvelinus* sp., GCF_002910315.2, *n* = 46,775) using the same annotation pipeline. However, it is important to note that annotation completeness is markedly reduced relative to other assemblies with similar BUSCO scores such as Atlantic Salmon (57,783; GCF_000233375.1; Annotation Release 100), Coho Salmon (63,465; GCF_002021735.2; Annotation Release 101), Brown Trout (61,583; GCF_901001165.1; Annotation Release 100), Rainbow Trout (55,630, GCF_002163495.1, Annotation Release 100), and Chinook Salmon (53,685, GCF_002872995.1, Annotation Release 100). These annotations were produced using RNA‐seq evidence from a greater diversity of tissue types, which probably explains this discrepancy. The Lake Trout annotation, as well as annotations for other salmonids, could also be further improved by directly sequencing full length transcripts using long‐read sequencing technologies (Workman et al., 2019). We predict that the completeness of the Lake Trout genome annotation will be improved as more gene expression data from a greater diversity of tissue types becomes available for the species (Salzberg, 2019). Nonetheless, the current genome annotation will undoubtably aid in the interpretation of future findings by allowing researchers to link signals of selection and loci associated with phenotypes with putatively causal genes and biological processes. Publicly available gene expression and functional annotation resources, like those being developed by the functional annotation of all salmonid genomes (FAASG) initiative, will also aid in this effort (Macqueen et al., 2017).

The availability of a second high‐quality genome assembly for a *Salvelinus* species will probably benefit comparative genomic research aimed at understanding the evolutionary consequences of genome duplication. Salmonids have long been appreciated as a model system for understanding evolution following whole genome duplication (Ohno, 1970) and a variety of recent studies have utilized the wealth of genomic resources for salmonids to shed light on the evolutionary processes at play following autotetraploid genome duplication events (see Gillard et al., 2021; Gundappa et al., 2021). Additionally, multiple recent studies have highlighted the importance of structural genetic variation for promoting adaptive diversification within salmonid species (Bertolotti et al., 2020; Pearse et al., 2019), and chromosome‐anchored genome assemblies are typically needed for detecting and genotyping structural variants (Mérot et al., 2020).

Genomic methods have dramatically increased the precision of population genetic analyses and have enabled researchers to address qualitatively unique questions that require some knowledge of genome structure and function (Waples et al., 2020). The genome assembly presented here will enable researchers to identify loci and candidate genes associated with phenotypic differentiation and reproductive isolation among Lake Trout ecotypes. Additionally, this resource will allow for the identification of loci associated with variation in fitness between Lake Trout hatchery strains in contemporary Great Lakes environments (Larson et al., 2021; Scribner et al., 2018) and loci that are adaptively diverged between hatchery strains. This information could help fisheries managers to maximize adaptive genetic diversity in re‐emerging wild populations and prioritize hatchery populations for continued propagation. Overall, the availability of a high‐quality reference genome for Lake Trout will probably have important implications for ongoing conservation projects in the Great Lakes region and elsewhere.

## CONFLICT OF INTEREST

The authors declare no conflicts of interest.

## AUTHOR CONTRIBUTIONS

S.R.S. drafted the manuscript and assisted with genome assembly and scaffolding. G.L. and K.T.S. assisted with drafting the manuscript, provided funding support, and helped initiate the project. L.B. and A.M.M. oversaw all components of the project, provided funding support, and assisted with drafting the manuscript. C.C.W. coordinated creation of double haploid individuals, assisted with manuscript development, and oversaw the generation of RNA sequencing data. E.N. assisted with genome scaffolding, finishing, and assembly validation. J.R. coordinated and supervised long‐read sequencing and Hi‐C data generation and coordinated data analysis. P.B. carried out library preparation and PacBio sequencing. H.D. carried out long‐read data quality control and long‐read contig assembly. P.M.N. carried out Hi‐C scaffolding. C.M.P. generated RNA sequencing data and assisted with drafting the manuscript.

## Supporting information

Supplementary Material

Appendix S1

## Data Availability

The Lake Trout whole genome assembly project has been deposited at DDBJ/ENA/GenBank under the accession JAEAGN000000000. The version described in this manuscript is version JAEAGN010000000. The GenBank assembly accession is GCA_016432855.1. RNA sequencing data generated for annotation is available in Sequence Read Archives associated with BioProject PRJNA682236. Pacific Biosciences long‐read data are associated with SRA accession SRS9083103. Illumina paired‐end reads for the Seneca strain diploid female are associated with SRA accession SRS9197136. Hi‐C data for muscle tissue from a diploid Killala strain female are associated with SRA accession SRS9186379. Code used for contig assembly, scaffolding, gap filling and polishing is available at https://github.com/smithsr90/LakeTroutGenome. All references to the Lake Trout genome annotation are in reference to annotation release 100. A complete record of data sources used for annotation is available at: https://www.ncbi.nlm.nih.gov/genome/annotation_euk/Salvelinus_namaycush/100/.

## References

[men13483-bib-0001] Allendorf, F. W. , & Danzmann, R. G. (1997). Secondary tetrasomic segregation of MDH‐B and preferential pairing of homeologues in rainbow trout. Genetics, 145(4), 1083–1092. 9093860 10.1093/genetics/145.4.1083 PMC1207878

[men13483-bib-0002] Allendorf, F. W. , & Thorgaard, G. H. (1984). Tetraploidy and the evolution of salmonid fishes. In B. Turner (Ed.), Evolutionary genetics of fishes (pp. 1–53). Springer.

[men13483-bib-0003] Balon, E. K. (1980). Charrs, salmonid fishes of the genus Salvelinus. Kluwer.

[men13483-bib-0004] Bankevich, A. , Nurk, S. , Antipov, D. , Gurevich, A. A. , Dvorkin, M. , Kulikov, A. S. , Lesin, V. M. , Nikolenko, S. I. , Pham, S. , Prjibelski, A. D. , Pyshkin, A. V. , Sirotkin, A. V. , Vyahhi, N. , Tesler, G. , Alekseyev, M. A. , & Pevzner, P. A. (2012). SPAdes: a new genome assembly algorithm and its applications to single‐cell sequencing. Journal of Computational Biology, 19(5), 455–477. 10.1089/cmb.2012.0021 22506599 PMC3342519

[men13483-bib-0005] Benaglia, T. , Chauveau, D. , Hunter, D. R. , & Young, D. (2009). mixtools: An R package for analyzing finite mixture models. Journal of Statistical Software, 32(6), 1–29.

[men13483-bib-0006] Berthelot, C. , Brunet, F. , Chalopin, D. , Juanchich, A. , Bernard, M. , Noël, B. , Bento, P. , Da Silva, C. , Labadie, K. , Alberti, A. , Aury, J.‐M. , Louis, A. , Dehais, P. , Bardou, P. , Montfort, J. , Klopp, C. , Cabau, C. , Gaspin, C. , Thorgaard, G. H. , … Guiguen, Y. (2014). The rainbow trout genome provides novel insights into evolution after whole‐genome duplication in vertebrates. Nature Communications, 5(1), 1–10. 10.1038/ncomms4657 PMC4071752 24755649

[men13483-bib-0007] Bertolotti, A. C. , Layer, R. M. , Gundappa, M. K. , Gallagher, M. D. , Pehlivanoglu, E. , Nome, T. , Robledo, D. , Kent, M. P. , Røsæg, L. L. , Holen, M. M. , Mulugeta, T. D. , Ashton, T. J. , Hindar, K. , Sægrov, H. , Florø‐Larsen, B. , Erkinaro, J. , Primmer, C. R. , Bernatchez, L. , Martin, S. A. M. , … Macqueen, D. J. (2020). The structural variation landscape in 492 Atlantic salmon genomes. Nature Communications, 11(1), 1–16. 10.1038/s41467-020-18972-x PMC7560756 33056985

[men13483-bib-0008] Blackie, C. T. , Weese, D. J. , & Noakes, D. L. G. (2003). Evidence for resource polymorphism in the lake charr (*Salvelinus namaycush*) population of Great Bear Lake, Northwest Territories. Canada. Ecoscience, 10(4), 509–514.

[men13483-bib-0009] Bourgey, M. , Dali, R. , Eveleigh, R. , Chen, K. C. , Letourneau, L. , Fillon, J. , Michaud, M. , Caron, M. , Sandoval, J. , Lefebvre, F. , Leveque, G. , Mercier, E. , Bujold, D. , Marquis, P. , Van, P. T. , Anderson de Lima Morais, D. , Tremblay, J. , Shao, X. , Henrion, E. , … Bourque, G. (2019). GenPipes: An open‐source framework for distributed and scalable genomic analyses. GigaScience, 8(6), giz037. 10.1093/gigascience/giz037 31185495 PMC6559338

[men13483-bib-0010] Catchen, J. , Amores, A. , & Bassham, S. (2020). Chromonomer: a tool set for repairing and enhancing assembled genomes through integration of genetic maps and conserved synteny. G3: Genes, Genomes, Genetics, 10(11), 4115–4128. 32912931 10.1534/g3.120.401485 PMC7642942

[men13483-bib-0011] Chaisson, M. J. , & Tesler, G. (2012). Mapping single molecule sequencing reads using basic local alignment with successive refinement (BLASR): Application and theory. BMC Bioinformatics, 13(1), 1–18. 10.1186/1471-2105-13-238 22988817 PMC3572422

[men13483-bib-0012] Chavarie, L. , Howland, K. , Harris, L. , & Tonn, W. (2015). Polymorphism in Lake Trout in Great Bear Lake: intra‐lake morphological diversification at two spatial scales. Biological Journal of the Linnean Society, 114(1), 109–125. 10.1111/bij.12398

[men13483-bib-0013] Chin, C.‐S. , Alexander, D. H. , Marks, P. , Klammer, A. A. , Drake, J. , Heiner, C. , Clum, A. , Copeland, A. , Huddleston, J. , Eichler, E. E. , Turner, S. W. , & Korlach, J. (2013). Nonhybrid, finished microbial genome assemblies from long‐read SMRT sequencing data. Nature Methods, 10(6), 563. 10.1038/nmeth.2474 23644548

[men13483-bib-0014] Chin, C.‐S. , Peluso, P. , Sedlazeck, F. J. , Nattestad, M. , Concepcion, G. T. , Clum, A. , Dunn, C. , O'Malley, R. , Figueroa‐Balderas, R. , Morales‐Cruz, A. , Cramer, G. R. , Delledonne, M. , Luo, C. , Ecker, J. R. , Cantu, D. , Rank, D. R. , & Schatz, M. C. (2016). Phased diploid genome assembly with single‐molecule real‐time sequencing. Nature Methods, 13(12), 1050–1054. 10.1038/nmeth.4035 27749838 PMC5503144

[men13483-bib-0015] Chomczynski, P. , & Sacchi, N. (2006). The single‐step method of RNA isolation by acid guanidinium thiocyanate–phenol–chloroform extraction: twenty‐something years on. Nature Protocols, 1(2), 581–585. 10.1038/nprot.2006.83 17406285

[men13483-bib-0016] Christensen, K. A. , Leong, J. S. , Sakhrani, D. , Biagi, C. A. , Minkley, D. R. , Withler, R. E. , Rondeau, E. B. , Koop, B. F. , & Devlin, R. H. (2018). Chinook salmon (*Oncorhynchus tshawytscha*) genome and transcriptome. PLoS One, 13(4), e0195461. 10.1371/journal.pone.0195461 29621340 PMC5886536

[men13483-bib-0017] Christensen, K. A. , Rondeau, E. B. , Minkley, D. R. , Leong, J. S. , Nugent, C. M. , Danzmann, R. G. , Ferguson, M. M. , Stadnik, A. , Devlin, R. H. , Muzzerall, R. , Edwards, M. , Davidson, W. S. , & Koop, B. F. (2018). The Arctic Char (*Salvelinus alpinus*) genome and transcriptome assembly. PLoS One, 13(9), e0204076. 30212580 10.1371/journal.pone.0204076 PMC6136826

[men13483-bib-0018] Christensen, K. A. , Rondeau, E. B. , Minkley, D. R. , Leong, J. S. , Nugent, C. M. , Danzmann, R. G. , Ferguson, M. M. , Stadnik, A. , Devlin, R. H. , Muzzerall, R. , Edwards, M. , Davidson, W. S. , & Koop, B. F. (2021). Retraction: The Arctic charr (*Salvelinus alpinus*) genome and transcriptome assembly. PLoS One, 16(2), e0247083. 33561157 10.1371/journal.pone.0247083 PMC7872265

[men13483-bib-0019] De‐Kayne, R. , Zoller, S. , & Feulner, P. G. (2020). A de novo chromosome‐level genome assembly of *Coregonus* sp. “Balchen”: One representative of the Swiss Alpine whitefish radiation. Molecular Ecology Resources, 20(4), 1093–1109. 32395896 10.1111/1755-0998.13187 PMC7497118

[men13483-bib-0020] Döring, A. , Weese, D. , Rausch, T. , & Reinert, K. (2008). SeqAn an efficient, generic C++ library for sequence analysis. BMC Bioinformatics, 9(1), 1–9. 10.1186/1471-2105-9-11 18184432 PMC2246154

[men13483-bib-0021] Du, K. , Stöck, M. , Kneitz, S. , Klopp, C. , Woltering, J. M. , Adolfi, M. C. , Feron, R. , Prokopov, D. , Makunin, A. , Kichigin, I. , Schmidt, C. , Fischer, P. , Kuhl, H. , Wuertz, S. , Gessner, J. , Kloas, W. , Cabau, C. , Iampietro, C. , Parrinello, H. , … Schartl, M. (2020). The sterlet sturgeon genome sequence and the mechanisms of segmental rediploidization. Nature Ecology & Evolution, 4(6), 841–852. 10.1038/s41559-020-1166-x 32231327 PMC7269910

[men13483-bib-0022] English, A. C. , Richards, S. , Han, Y. I. , Wang, M. , Vee, V. , Qu, J. , Qin, X. , Muzny, D. M. , Reid, J. G. , Worley, K. C. , & Gibbs, R. A. (2012). Mind the gap: Upgrading genomes with Pacific Biosciences RS long‐read sequencing technology. PLoS One, 7(11), e47768. 10.1371/journal.pone.0047768 23185243 PMC3504050

[men13483-bib-0023] Fitzsimons, J. D. , Brown, S. B. , Williston, B. , Williston, G. , Brown, L. R. , Moore, K. , Honeyfield, D. C. , & Tillitt, D. E. (2009). Influence of thiamine deficiency on Lake Trout larval growth, foraging, and predator avoidance. Journal of Aquatic Animal Health, 21(4), 302–314. 10.1577/H08-019.1 20218504

[men13483-bib-0024] Flynn, J. M. , Hubley, R. , Goubert, C. , Rosen, J. , Clark, A. G. , Feschotte, C. , & Smit, A. F. (2020). RepeatModeler2 for automated genomic discovery of transposable element families. Proceedings of the National Academy of Sciences of the United States of America, 117(17), 9451–9457. 10.1073/pnas.1921046117 32300014 PMC7196820

[men13483-bib-0025] Gagnaire, P. A. , Normandeau, E. , Pavey, S. A. , & Bernatchez, L. (2013). Mapping phenotypic, expression and transmission ratio distortion QTL using RAD markers in the Lake Whitefish (*Coregonus clupeaformis*). Molecular Ecology, 22(11), 3036–3048. 23181719 10.1111/mec.12127

[men13483-bib-0026] Garrison, E. , & Marth, G. (2012). Haplotype‐based variant detection from short‐read sequencing. arXiv. preprint arXiv:1207.3907.

[men13483-bib-0027] Ghurye, J. , Pop, M. , Koren, S. , Bickhart, D. , & Chin, C. S. (2017). Scaffolding of long read assemblies using long range contact information. BMC Genomics, 18(1), 1–11. 10.1186/s12864-017-3879-z 28701198 PMC5508778

[men13483-bib-0028] Gillard, G. B. , Grønvold, L. , Røsæg, L. L. , Holen, M. M. , Monsen, Ø. , Koop, B. F. , Rondeau, E. B. , Gundappa, M. K. , Mendoza, J. , Macqueen, D. J. , Rohlfs, R. V. , Sandve, S. R. , & Hvidsten, T. R. (2021). Comparative regulomics supports pervasive selection on gene dosage following whole genome duplication. Genome Biology, 22(1), 1–18. 10.1186/s13059-021-02323-0 33849620 PMC8042706

[men13483-bib-0029] Goetz, F. , Rosauer, D. , Sitar, S. , Goetz, G. , Simchick, C. , Roberts, S. , & Mackenzie, S. (2010). A genetic basis for the phenotypic differentiation between siscowet and lean Lake Trout (*Salvelinus namaycush*). Molecular Ecology, 19, 176–196. 20331779 10.1111/j.1365-294X.2009.04481.x

[men13483-bib-0030] Goetz, F. , Smith, S. E. , Goetz, G. , & Murphy, C. A. (2016). Sea lampreys elicit strong transcriptomic responses in the Lake Trout liver during parasitism. BMC Genomics, 17(1), 1–16. 10.1186/s12864-016-2959-9 27558222 PMC4997766

[men13483-bib-0031] Goodier, J. L. (1981). Native Lake Trout (*Salvelinus namaycush*) stocks in the Canadian waters of Lake Superior prior to 1955. Canadian Journal of Fisheries and Aquatic Sciences, 38(12), 1724–1737.

[men13483-bib-0032] Gu, Z. , Gu, L. , Eils, R. , Schlesner, M. , & Brors, B. (2014). circlize implements and enhances circular visualization in R. Bioinformatics, 30(19), 2811–2812. 10.1093/bioinformatics/btu393 24930139

[men13483-bib-0033] Gundappa, M. K. , To, T. , Grønvold, L. , Martin, S. , Lien, S. , Geist, J. , Hazlerigg, D. , Sandve, S. , & Macqueen, D. (2021). Genome‐wide reconstruction of rediploidization following autopolyploidization across one hundred million years of salmonid evolution. bioRxiv. 10.1093/molbev/msab310 PMC8760942 34718723

[men13483-bib-0034] Haas, B. J. , Delcher, A. L. , Wortman, J. R. , & Salzberg, S. L. (2004). DAGchainer: A tool for mining segmental genome duplications and synteny. Bioinformatics, 20(18), 3643–3646. 10.1093/bioinformatics/bth397 15247098

[men13483-bib-0035] Hansen, M. J. (1999). Lake trout in the Great Lakes: Basin‐wide stock collapse and binational restoration. In W. W. Taylor & C. P. Ferreri (Eds.), Great lakes fishery policy and management: A binational perspective (pp. 417–453). Michigan State University Press.

[men13483-bib-0036] Hansen, M. J. , Nate, N. A. , Muir, A. M. , Bronte, C. R. , Zimmerman, M. S. , & Krueger, C. C. (2016). Life history variation among four Lake Trout morphs at Isle Royale, Lake Superior. Journal of Great Lakes Research, 42(2), 421–432. 10.1016/j.jglr.2015.12.011

[men13483-bib-0037] Hansen, T. J. , Penman, D. , Glover, K. A. , Fraser, T. W. K. , Vågseth, T. , Thorsen, A. , & Fjelldal, P. G. (2020). Production and verification of the first Atlantic salmon (*Salmo salar* L.) clonal lines. BMC Genetics, 21(1), 1–10. 32641046 10.1186/s12863-020-00878-8 PMC7346428

[men13483-bib-0038] Hanson, S. D. , Holey, M. E. , Treska, T. J. , Bronte, C. R. , & Eggebraaten, T. H. (2013). Evidence of wild juvenile Lake Trout recruitment in western Lake Michigan. North American Journal of Fisheries Management, 33(1), 186–191. 10.1080/02755947.2012.754804

[men13483-bib-0039] Harris, L. N. , Chavarie, L. , Bajno, R. , Howland, K. L. , Wiley, S. H. , Tonn, W. M. , & Taylor, E. B. (2015). Evolution and origin of sympatric shallow‐water morphotypes of Lake Trout, *Salvelinus namaycush*, in Canada’s Great Bear Lake. Heredity, 114(1), 94–106. 10.1038/hdy.2014.74 25204304 PMC4815598

[men13483-bib-0040] Haug‐Baltzell, A. , Stephens, S. A. , Davey, S. , Scheidegger, C. E. , & Lyons, E. (2017). SynMap2 and SynMap3D: Web‐based whole‐genome synteny browsers. Bioinformatics, 33(14), 2197–2198. 10.1093/bioinformatics/btx144 28334338

[men13483-bib-0041] Hotaling, S. , & Kelley, J. L. (2020). The rising tide of high‐quality genomic resources. Molecular Ecology Resources, 19, 567–569. 10.1111/1755-0998.12964 31004471

[men13483-bib-0042] Kajitani, R. , Toshimoto, K. , Noguchi, H. , Toyoda, A. , Ogura, Y. , Okuno, M. , Yabana, M. , Harada, M. , Nagayasu, E. , Maruyama, H. , Kohara, Y. , Fujiyama, A. , Hayashi, T. , & Itoh, T. (2014). Efficient de novo assembly of highly heterozygous genomes from whole‐genome shotgun short reads. Genome Research, 24(8), 1384–1395. 10.1101/gr.170720.113 24755901 PMC4120091

[men13483-bib-0043] Kodama, M. , Brieuc, M. S. , Devlin, R. H. , Hard, J. J. , & Naish, K. A. (2014). Comparative mapping between Coho Salmon (*Oncorhynchus kisutch*) and three other salmonids suggests a role for chromosomal rearrangements in the retention of duplicated regions following a whole genome duplication event. G3: Genes, Genomes, Genetics, 4(9), 1717–1730. 25053705 10.1534/g3.114.012294 PMC4169165

[men13483-bib-0044] Komen, H. , & Thorgaard, G. H. (2007). Androgenesis, gynogenesis and the production of clones in fishes: A review. Aquaculture, 269(1–4), 150–173. 10.1016/j.aquaculture.2007.05.009

[men13483-bib-0045] Koren, S. , Walenz, B. P. , Berlin, K. , Miller, J. R. , Bergman, N. H. , & Phillippy, A. M. (2017). Canu: Scalable and accurate long‐read assembly via adaptive k‐mer weighting and repeat separation. Genome Research, 27(5), 722–736. 28298431 10.1101/gr.215087.116 PMC5411767

[men13483-bib-0046] Krueger, C. C. , Horrall, R. M. , & Gruenthal, H. (1983). Strategy for the use of Lake Trout strains in Lake Michigan. Wisconsin Department of Natural Resources, Administrative Report, 17.

[men13483-bib-0047] Krueger, C. C. , & Ihssen, P. E. (1995). Review of genetics of Lake Trout in the Great Lakes: History, molecular genetics, physiology, strain comparisons, and restoration management. Journal of Great Lakes Research, 21, 348–363. 10.1016/S0380-1330(95)71109-1

[men13483-bib-0048] Lantry, J. R. (2015). Eastern basin of Lake Ontario warmwater fisheries assessment, 1976–2014. In 2014 annual report, Bureau of Fisheries, Lake Ontario Unit and St Lawrence River Unit to the Great Lakes Fishery Commission’s Lake Ontario Committee (pp. 1–35).

[men13483-bib-0049] Larson, W. , Kornis, M. , Turnquist, K. N. , Bronte, C. , Holey, M. E. , Hanson, D. , Treska, T. , Stott, W. , & Sloss, B. (2021). The genetic composition of wild recruits in a recovering Lake Trout population in Lake Michigan. Canadian Journal of Fisheries and Aquatic Sciences, 99(999), 1–15.

[men13483-bib-0050] Li, H. (2013). Aligning sequence reads, clone sequences and assembly contigs with BWA‐MEM. arXiv preprint arXiv:1303.3997.

[men13483-bib-0051] Li, H. (2018). Minimap2: Pairwise alignment for nucleotide sequences. Bioinformatics, 34(18), 3094–3100. 10.1093/bioinformatics/bty191 29750242 PMC6137996

[men13483-bib-0052] Li, H. , Handsaker, B. , Wysoker, A. , Fennell, T. , Ruan, J. , Homer, N. , Marth, G. , Abecasis, G. , & Durbin, R. (2009). The sequence alignment/map format and SAMtools. Bioinformatics, 25(16), 2078–2079. 10.1093/bioinformatics/btp352 19505943 PMC2723002

[men13483-bib-0053] Lien, S. , Koop, B. , Sandve, S. , Miller, J. R. , Kent, M. , Nome, T. , Hvidsten, T. R. , Leong, J. , Minkley, D. , Zimin, A. , Grammes, F. , Grove, H. , Gjuvsland, A. , Walenz, B. , Hermansen, R. A. , Schalburg, K. V. , Rondeau, E. , Génova, A. , Samy, J. K. , … Davidson, W. (2016). The Atlantic salmon genome provides insights into rediploidization. Nature, 533(7602), 200–205. 27088604 10.1038/nature17164 PMC8127823

[men13483-bib-0054] Limborg, M. T. , Seeb, L. W. , & Seeb, J. E. (2016). Sorting duplicated loci disentangles complexities of polyploid genomes masked by genotyping by sequencing. Molecular Ecology, 25(10), 2117–2129. 10.1111/mec.13601 26939067

[men13483-bib-0055] Lynch, M. , & Force, A. G. (2000). The origin of interspecific genomic incompatibility via gene duplication. The American Naturalist, 156(6), 590–605. 10.1086/316992 29592543

[men13483-bib-0056] Mac, M. J. , & Edsall, C. C. (1991). Environmental contaminants and the reproductive success of Lake Trout in the Great Lakes: An epidemiological approach. Journal of Toxicology and Environmental Health, Part A Current Issues, 33(4), 375–394. 10.1080/15287399109531536 1908524

[men13483-bib-0057] Macqueen, D. J. , & Johnston, I. A. (2014). A well‐constrained estimate for the timing of the salmonid whole genome duplication reveals major decoupling from species diversification. Proceedings of the Royal Society B: Biological Sciences, 281(1778), 20132881. 10.1098/rspb.2013.2881 PMC3906940 24452024

[men13483-bib-0058] Macqueen, D. J. , Primmer, C. R. , Houston, R. D. , Nowak, B. F. , Bernatchez, L. , Bergseth, S. , Davidson, W. S. , Gallardo‐Escárate, C. , Goldammer, T. , Guiguen, Y. , Iturra, P. , Kijas, J. W. , Koop, B. F. , Lien, S. , Maass, A. , Martin, S. A. M. , McGinnity, P. , Montecino, M. , Naish, K. A. , … Yáñez, J. M. (2017). Functional Annotation of All Salmonid Genomes (FAASG): An international initiative supporting future salmonid research, conservation and aquaculture. BMC Genomics, 18(1), 1–9. 28655320 10.1186/s12864-017-3862-8 PMC5488370

[men13483-bib-0059] Madenjian, C. P. , O'Gorman, R. , Bunnell, D. B. , Argyle, R. L. , Roseman, E. F. , Warner, D. M. , Stockwell, J. D. , & Stapanian, M. A. (2008). Adverse effects of alewives on Laurentian Great Lakes fish communities. North American Journal of Fisheries Management, 28(1), 263–282. 10.1577/M07-012.1

[men13483-bib-0060] Marçais, G. , Delcher, A. L. , Phillippy, A. M. , Coston, R. , Salzberg, S. L. , & Zimin, A. (2018). MUMmer4: A fast and versatile genome alignment system. PLoS Computational Biology, 14(1), e1005944. 10.1371/journal.pcbi.1005944 29373581 PMC5802927

[men13483-bib-0061] Marçais, G. , & Kingsford, C. (2011). A fast, lock‐free approach for efficient parallel counting of occurrences of k‐mers. Bioinformatics, 27(6), 764–770. 10.1093/bioinformatics/btr011 21217122 PMC3051319

[men13483-bib-0062] Marin, K. , Coon, A. , Carson, R. , Debes, P. V. , & Fraser, D. J. (2016). Striking phenotypic variation yet low genetic differentiation in sympatric Lake Trout (*Salvelinus namaycush*). PLoS One, 11(9), e0162325. 10.1371/journal.pone.0162325 27680019 PMC5040267

[men13483-bib-0063] Marsden, J. E. , Noakes, D. L. , & Krueger, C. C. (2021). Terminology Issues in Lake Charr Early Development. In A. M. Muir (Ed.), The Lake Charr *Salvelinus namaycush*: Biology, ecology, distribution, and management (pp. 487–497). Springer International Publishing.

[men13483-bib-0064] Mérot, C. , Oomen, R. A. , Tigano, A. , & Wellenreuther, M. (2020). A roadmap for understanding the evolutionary significance of structural genomic variation. Trends in Ecology & Evolution, 35(7), 561–572. 10.1016/j.tree.2020.03.002 32521241

[men13483-bib-0065] Muir, A. M. , Bronte, C. R. , Zimmerman, M. S. , Quinlan, H. R. , Glase, J. D. , & Krueger, C. C. (2014). Ecomorphological diversity of Lake Trout at Isle Royale, Lake Superior. Transactions of the American Fisheries Society, 143(4), 972–987. 10.1080/00028487.2014.900823

[men13483-bib-0066] Muir, A. M. , Hansen, M. J. , Bronte, C. R. , & Krueger, C. C. (2016). If Arctic charr *Salvelinus alpinus* is ‘the most diverse vertebrate’, what is the lake charr *Salvelinus namaycush*? Fish and Fisheries, 17(4), 1194–1207.

[men13483-bib-0067] Ohno, S. (1970). Evolution by gene duplication. Springer‐Verlag.

[men13483-bib-0068] Page, K. S. , Scribner, K. T. , Bennett, K. R. , Garzel, L. M. , & Burnham‐Curtis, M. K. (2003). Genetic assessment of strain‐specific sources of Lake Trout recruitment in the Great Lakes. Transactions of the American Fisheries Society, 132(5), 877–894. 10.1577/T02-092

[men13483-bib-0069] Pan, W. , Jiang, T. , & Lonardi, S. (2020). OMGS: Optical map‐based genome scaffolding. Journal of Computational Biology, 27(4), 519–533. 10.1089/cmb.2019.0310 31794680

[men13483-bib-0070] Pearse, D. E. , Barson, N. J. , Nome, T. , Gao, G. , Campbell, M. A. , Abadía‐Cardoso, A. , Anderson, E. C. , Rundio, D. E. , Williams, T. H. , Naish, K. A. , Moen, T. , Liu, S. , Kent, M. , Moser, M. , Minkley, D. R. , Rondeau, E. B. , Brieuc, M. S. O. , Sandve, S. R. , Miller, M. R. , … Lien, S. (2019). Sex‐dependent dominance maintains migration supergene in rainbow trout. Nature Ecology & Evolution, 3(12), 1731–1742. 10.1038/s41559-019-1044-6 31768021

[men13483-bib-0071] Perkins, D. L. , Fitzsimons, J. D. , Marsden, J. E. , Krueger, C. C. , & May, B. (1995). Differences in reproduction among hatchery strains of Lake Trout at eight spawning areas in Lake Ontario: Genetic evidence from mixed‐stock analysis. Journal of Great Lakes Research, 21, 364–374. 10.1016/S0380-1330(95)71110-8

[men13483-bib-0072] Pflug, J. M. , Holmes, V. R. , Burrus, C. , Johnston, J. S. , & Maddison, D. R. (2020). Measuring genome sizes using read‐depth, k‐mers, and flow cytometry: Methodological comparisons in beetles (Coleoptera). G3: Genes, Genomes, Genetics, 10(9), 3047–3060. 32601059 10.1534/g3.120.401028 PMC7466995

[men13483-bib-0073] Prince, D. J. , O’Rourke, S. M. , Thompson, T. Q. , Ali, O. A. , Lyman, H. S. , Saglam, I. K. , Hotaling, T. J. , Spidle, A. P. , & Miller, M. R. (2017). The evolutionary basis of premature migration in Pacific salmon highlights the utility of genomics for informing conservation. Science Advances, 3(8), e1603198. 10.1126/sciadv.1603198 28835916 PMC5559211

[men13483-bib-0074] Pycha, R. L. (1980). Changes in mortality of Lake Trout (*Salvelinus namaycush*) in Michigan waters of Lake Superior in relation to sea lamprey (*Petromyzon marinus*) predation, 1968–78. Canadian Journal of Fisheries and Aquatic Sciences, 37(11), 2063–2073.

[men13483-bib-0075] Quinlan, A. R. , & Hall, I. M. (2010). BEDTools: A flexible suite of utilities for comparing genomic features. Bioinformatics, 26(6), 841–842. 10.1093/bioinformatics/btq033 20110278 PMC2832824

[men13483-bib-0076] R Core Team (2017). R: A language and environment for statistical computing. R Foundation for Statistical Computing. https://www.R‐project.org/

[men13483-bib-0077] Rezvoy, C. , Charif, D. , Guéguen, L. , & Marais, G. A. (2007). MareyMap: An R‐based tool with graphical interface for estimating recombination rates. Bioinformatics, 23(16), 2188–2189. 10.1093/bioinformatics/btm315 17586550

[men13483-bib-0078] Riley, S. C. , He, J. X. , Johnson, J. E. , O’Brien, T. P. , & Schaeffer, J. S. (2007). Evidence of widespread natural reproduction by Lake Trout i in the Michigan waters of Lake Huron. Journal of Great Lakes Research, 33(4), 917–921. 10.3394/0380-1330(2007)33#;917:EOWNRB#;2.0.CO;2

[men13483-bib-0079] Robertson, F. M. , Gundappa, M. K. , Grammes, F. , Hvidsten, T. R. , Redmond, A. K. , Lien, S. , Martin, S. A. M. , Holland, P. W. H. , Sandve, S. R. , & Macqueen, D. J. (2017). Lineage‐specific rediploidization is a mechanism to explain time‐lags between genome duplication and evolutionary diversification. Genome Biology, 18(1), 1–14. 10.1186/s13059-017-1241-z 28615063 PMC5470254

[men13483-bib-0080] Rondeau, E. B. , Minkley, D. R. , Leong, J. S. , Messmer, A. M. , Jantzen, J. R. , von Schalburg, K. R. , Lemon, C. , Bird, N. H. , & Koop, B. F. (2014). The genome and linkage map of the northern pike (*Esox lucius*): Conserved synteny revealed between the salmonid sister group and the Neoteleostei. PLoS One, 9(7), e102089. 10.1371/journal.pone.0102089 25069045 PMC4113312

[men13483-bib-0081] Rougeux, C. , Gagnaire, P. A. , Praebel, K. , Seehausen, O. , & Bernatchez, L. (2019). Polygenic selection drives the evolution of convergent transcriptomic landscapes across continents within a Nearctic sister species complex. Molecular Ecology, 28(19), 4388–4403. 10.1111/mec.15226 31482603

[men13483-bib-0082] Ruan, J. , & Li, H. (2020). Fast and accurate long‐read assembly with wtdbg2. Nature Methods, 17(2), 155–158. 10.1038/s41592-019-0669-3 31819265 PMC7004874

[men13483-bib-0083] Salzberg, S. L. (2019). Next‐generation genome annotation: we still struggle to get it right. Genome Biology, 20, 92. 10.1186/s13059-019-1715-2. 31097009 PMC6521345

[men13483-bib-0084] Sambrook, J. , & Russell, D. W. (2001). Molecular cloning ‐Vol. 1, 2, 3. Cold Springs Harbour Laboratory Press.

[men13483-bib-0085] Schroeter, J. C. , Maloy, A. P. , Rees, C. B. , & Bartron, M. L. (2020). Fish mitochondrial genome sequencing: expanding genetic resources to support species detection and biodiversity monitoring using environmental DNA. Conservation Genetics Resources, 12(3), 433–446. 10.1007/s12686-019-01111-0

[men13483-bib-0086] Scribner, K. , Tsehaye, I. , Brenden, T. , Stott, W. , Kanefsky, J. , & Bence, J. (2018). Hatchery strain contributions to emerging wild Lake Trout populations in Lake Huron. Journal of Heredity, 109(6), 675–688. 10.1093/jhered/esy029 29924322

[men13483-bib-0087] Shedko, S. V. (2019). Assembly ASM291031v2 (Genbank: GCA_002910315. 2) identified as assembly of the Northern Dolly Varden (*Salvelinus malma malma*) genome, and not the Arctic char (*S. alpinus*) genome. arXiv preprint arXiv:1912.02474.

[men13483-bib-0088] Siberchicot, A. , Bessy, A. , Guéguen, L. , & Marais, G. A. (2017). MareyMap online: A user‐friendly web application and database service for estimating recombination rates using physical and genetic maps. Genome Biology and Evolution, 9(10), 2506–2509. 10.1093/gbe/evx178 28981643 PMC5737613

[men13483-bib-0089] Smit, A. F. A. , Hubley, R. , & Green, P. (2015). RepeatMasker Open‐4.0. http://www.repeatmasker.org

[men13483-bib-0090] Smith, S. H. (1968). Species succession and fishery exploitation in the Great Lakes. Journal of the Fisheries Research Board of Canada, 25, 667–693.

[men13483-bib-0091] Smith, S. R. , Amish, S. J. , Bernatchez, L. , Le Luyer, J. , Wilson, C. , Boeberitz, O. , Luikart, G. , & Scribner, K. T. (2020). Mapping of adaptive traits enabled by a high‐density linkage map for Lake Trout. G3: Genes, Genomes, Genetics, 10(6), 1929–1947. 10.1534/g3.120.401184 32284313 PMC7263693

[men13483-bib-0092] Soderlund, C. , Bomhoff, M. , & Nelson, W. M. (2011). SyMAP v3. 4: A turnkey synteny system with application to plant genomes. Nucleic Acids Research, 39(10), e68. 21398631 10.1093/nar/gkr123 PMC3105427

[men13483-bib-0093] Soderlund, C. , Nelson, W. , Shoemaker, A. , & Paterson, A. (2006). SyMAP: A system for discovering and viewing syntenic regions of FPC maps. Genome Research, 16(9), 1159–1168. 10.1101/gr.5396706 16951135 PMC1557773

[men13483-bib-0094] Tang, H. , Lyons, E. , Pedersen, B. , Schnable, J. C. , Paterson, A. H. , & Freeling, M. (2011). Screening synteny blocks in pairwise genome comparisons through integer programming. BMC Bioinformatics, 12(1), 1–11. 10.1186/1471-2105-12-102 21501495 PMC3088904

[men13483-bib-0095] Thibaud‐Nissen, F. , DiCuccio, M. , Hlavina, W. , Kimchi, A. , Kitts, P. A. , Murphy, T. D. , Pruitt, K. D. , & Souvorov, A. (2016). P8008 the NCBI eukaryotic genome annotation pipeline. Journal of Animal Science, 94(suppl_4), 184. 10.2527/jas2016.94supplement4184x

[men13483-bib-0096] Thorgaard, G. H. , Allendorf, F. W. , & Knudsen, K. L. (1983). Gene‐centromere mapping in rainbow trout: High interference over long map distances. Genetics, 103(4), 771–783. 10.1093/genetics/103.4.771 17246124 PMC1202053

[men13483-bib-0097] Valiquette, E. , Perrier, C. , Thibault, I. , & Bernatchez, L. (2014). Loss of genetic integrity in wild Lake Trout populations following stocking: Insights from an exhaustive study of 72 lakes from Québec. Canada. Evolutionary Applications, 7(6), 625–644. 25067947 10.1111/eva.12160 PMC4105915

[men13483-bib-0098] Van de Peer, Y. , Mizrachi, E. , & Marchal, K. (2017). The evolutionary significance of polyploidy. Nature Reviews Genetics, 18(7), 411. 10.1038/nrg.2017.26 28502977

[men13483-bib-0099] Veale, A. J. , & Russello, M. A. (2017). An ancient selective sweep linked to reproductive life history evolution in sockeye salmon. Scientific Reports, 7(1), 1–10. 10.1038/s41598-017-01890-2 28496186 PMC5431894

[men13483-bib-0100] Vurture, G. W. , Sedlazeck, F. J. , Nattestad, M. , Underwood, C. J. , Fang, H. , Gurtowski, J. , & Schatz, M. C. (2017). GenomeScope: Fast reference‐free genome profiling from short reads. Bioinformatics, 33(14), 2202–2204. 10.1093/bioinformatics/btx153 28369201 PMC5870704

[men13483-bib-0101] Waples, R. S. , Naish, K. A. , & Primmer, C. R. (2020). Conservation and management of salmon in the age of genomics. Annual Review of Animal Biosciences, 8, 117–143. 10.1146/annurev-animal-021419-083617 31730428

[men13483-bib-0102] Watson, M. , & Warr, A. (2019). Errors in long‐read assemblies can critically affect protein prediction. Nature Biotechnology, 37(2), 124–126. 10.1038/s41587-018-0004-z 30670796

[men13483-bib-0103] Whibley, A. , Kelley, J. , & Narum, S. (2020). The changing face of genome assemblies: Guidance on achieving high‐quality reference genomes. Molecular Ecology Resources, 21(3), 641–652. 10.1111/1755-0998.13312 33326691

[men13483-bib-0104] Wick, R. R. , Judd, L. M. , Gorrie, C. L. , & Holt, K. E. (2017). Unicycler: resolving bacterial genome assemblies from short and long sequencing reads. PLoS Computational Biology, 13(6), e1005595. 10.1371/journal.pcbi.1005595 28594827 PMC5481147

[men13483-bib-0105] Williams, D. , Trimble, W. L. , Shilts, M. , Meyer, F. , & Ochman, H. (2013). Rapid quantification of sequence repeats to resolve the size, structure and contents of bacterial genomes. BMC Genomics, 14(1), 1–11. 10.1186/1471-2164-14-537 23924250 PMC3751351

[men13483-bib-0106] Willoughby, J. R. , Harder, A. M. , Tennessen, J. A. , Scribner, K. T. , & Christie, M. R. (2018). Rapid genetic adaptation to a novel environment despite a genome‐wide reduction in genetic diversity. Molecular Ecology, 27(20), 4041–4051. 10.1111/mec.14726 29802799

[men13483-bib-0107] Wingett, S. , Ewels, P. , Furlan‐Magaril, M. , Nagano, T. , Schoenfelder, S. , Fraser, P. , & Andrews, S. (2015). HiCUP: pipeline for mapping and processing Hi‐C data. F1000Research, 4, 1310. 10.12688/f1000research.7334.1 26835000 PMC4706059

[men13483-bib-0108] Workman, R. E. , Tang, A. D. , Tang, P. S. , Jain, M. , Tyson, J. R. , Razaghi, R. , Zuzarte, P. C. , Gilpatrick, T. , Payne, A. , Quick, J. , Sadowski, N. , Holmes, N. , de Jesus, J. G. , Jones, K. L. , Soulette, C. M. , Snutch, T. P. , Loman, N. , Paten, B. , Loose, M. , … Timp, W. (2019). Nanopore native RNA sequencing of a human poly (A) transcriptome. Nature Methods, 16(12), 1297–1305. 10.1038/s41592-019-0617-2 31740818 PMC7768885

[men13483-bib-0109] Zimin, A. V. , & Salzberg, S. L. (2020). The genome polishing tool POLCA makes fast and accurate corrections in genome assemblies. PLoS Computational Biology, 16(6), e1007981. 10.1371/journal.pcbi.1007981 32589667 PMC7347232

[men13483-bib-0110] Zimmerman, M. S. , Krueger, C. C. , & Eshenroder, R. L. (2006). Phenotypic diversity of Lake Trout in Great Slave Lake: Differences in morphology, buoyancy, and habitat depth. Transactions of the American Fisheries Society, 135(4), 1056–1067. 10.1577/T05-237.1

