# Supplementary material for: A chromosome‐anchored genome assembly for Lake Trout (Salvelinus namaycush)
Source: Mol Ecol Resour. 2021 Aug 14;22(2):679–94. doi: 10.1111/1755-0998.13483 (PMC9291852; doi:10.1111/1755-0998.13483)
Supplement: Supplementary file 1 — Supplementary Material [file MEN-22-679-s002.docx]

**Supplemental Information for:**

**A chromosome-anchored genome assembly for Lake Trout (Salvelinus namaycush)**

Seth R. Smith, Eric Normandeau, Haig Djambazian, Pubudu M. Nawarathna, Pierre Berube, Andrew M. Muir, Jiannis Ragoussis, Chantelle M. Penney, Kim T. Scribner, Gordon Luikart, Chris C. Wilson, and Louis Bernatchez

**Table of Contents:**

| **Supplemental Table 1: GenomeScope Output** | Page 2 |
| --- | --- |
| **Supplemental Figure 1: GenomeScope Output** | Page 3 |
| **Supplemental Figure 2: Dolly Varden Synteny** | Page 4 |
| **Supplemental Figure 3: Atlantic Salmon Synteny** | Page 5 |
| **Supplemental Figure 4: Rainbow Trout Synteny** | Page 6 |
| **Supplemental Figure 5: Northern Pike Synteny** | Page 7 |
| **Supplemental Figure 6: Neighbor Joining Tree** | Page 8 |
| **Supplemental Table 2: BUSCO Comparison** | Page 9 |
| **Supplemental Table 3: N50 Comparison** | Page 10 |
| **Supplemental Table 4: Centromere Locations** | Page 11 |

Supplemental Table 1: GenomeScope Output

| **GenomeScope version 1.0** | |  |
| --- | --- | --- |
| **k = 19, Read Length = 150, Max Coverage = -1** | | |
| **Sample SLW_52_F** |  |  |
|  |  |  |
| **Property** | **Minimum** | **Maximum** |
| Heterozygosity | 0.278% | 0.290% |
| Genome Haploid Length | 2,119,589,342 | 2,122,166,134 |
| Genome Repeat Length | 1,316,156,520 | 1,317,756,576 |
| Genome Unique Length | 803,432,822 | 804,409,558 |
| Model Fit | 92.373% | 99.196% |
| Read Error Rate | 0.0288% | 0.0288% |

Supplemental Figure 1: GenomeScope Output


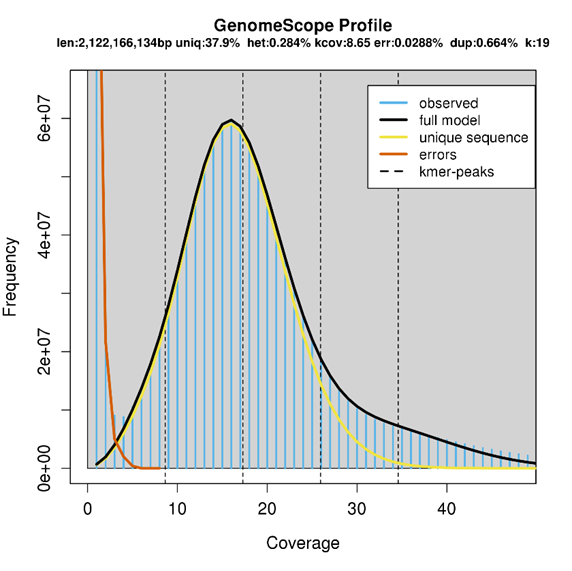


Supplemental Figure 2: Syntenic relationships between Lake Trout and Dolly Varden (previously Arctic Char) assemblies

The circos plot below identifies syntenic blocks shared between the Lake Trout and Dolly Varden genomes. Links are drawn between homologous regions in the two assemblies. Syntenic blocks were identified using SyMap version 5. Genomes were aligned using Promer and we used the Symap options min_dots = 30, top_n = 1, merge_blocks = 1, and no_overlapping_blocks = 1. The plot was generated using the Chromosome Explorer option in SyMap. A complete record of syntenic blocks between these two genomes is available in tab delimited format upon request.


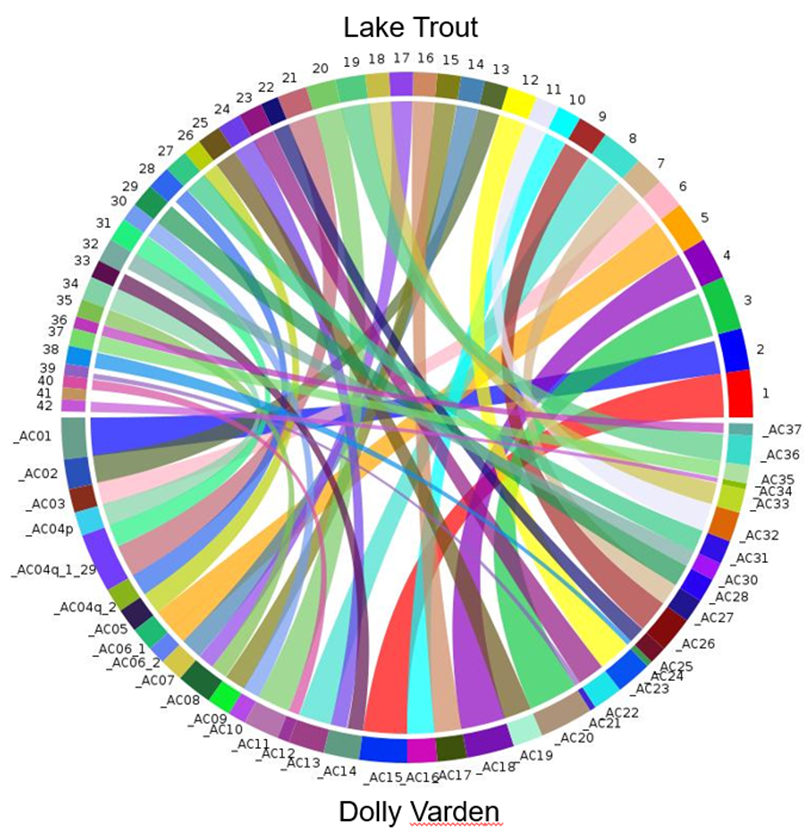


Supplemental Figure 3: Syntenic relationships between Lake Trout and Atlantic Salmon genome assemblies

The circos plot below identifies syntenic blocks shared between the Lake Trout and Atlantic Salmon genomes. Links are drawn between homologous regions in the two assemblies. Syntenic blocks were identified using SyMap version 5. Genomes were aligned using Promer and we used the Symap options min_dots = 30, top_n = 1, merge_blocks = 1, and no_overlapping_blocks = 1. The plot was generated using the Chromosome Explorer option in SyMap. A complete record of syntenic blocks between these two genomes is available in tab delimited format upon request.


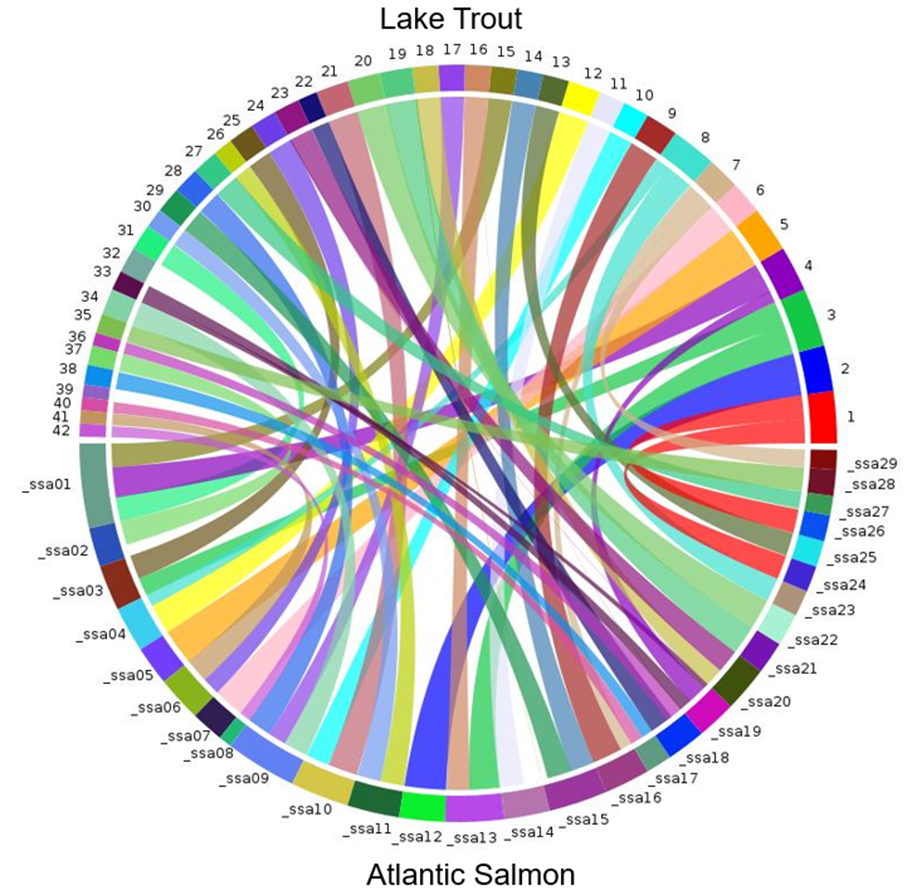


Supplemental Figure 4: Syntenic relationships between Lake Trout and Rainbow Trout genome assemblies

The circos plot below identifies syntenic blocks shared between the Lake Trout and Rainbow Trout genomes. Links are drawn between homologous regions in the two species. Syntenic blocks were identified using SyMap version 5. Genomes were aligned using Promer and we used the Symap options min_dots = 30, top_n = 1, merge_blocks = 1, and no_overlapping_blocks = 1. The plot was generated using the Chromosome Explorer option in SyMap. A complete record of syntenic blocks between these two genomes is available in tab delimited format upon request.


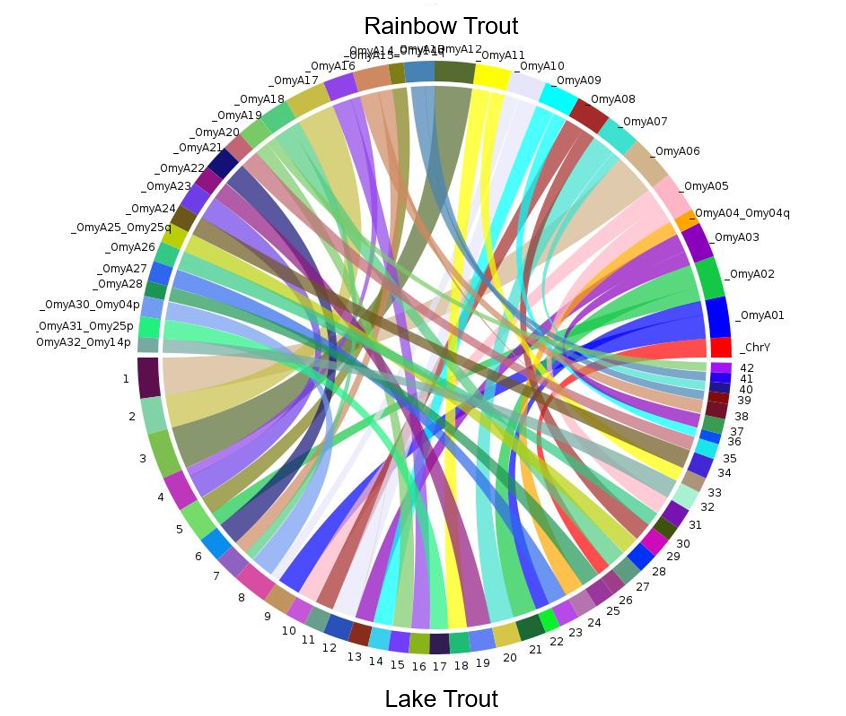


Supplemental Figure 5: Syntenic relationships between Lake Trout and Northern Pike genome assemblies

The dot plot below identifies syntenic blocks shared between the Lake Trout and Northern Pike genomes. Within SynMap2, we used the last algorithm to align genomes, DAGChainer to identify syntenic blocks (-D20, -A5), Quota Align Merge to merge syntenic blocks (-Dm 0), and Quota Align (Overlap Distance = 40) to enforce a 1-to-2 ploidy relationship between Northern Pike and Lake Trout.


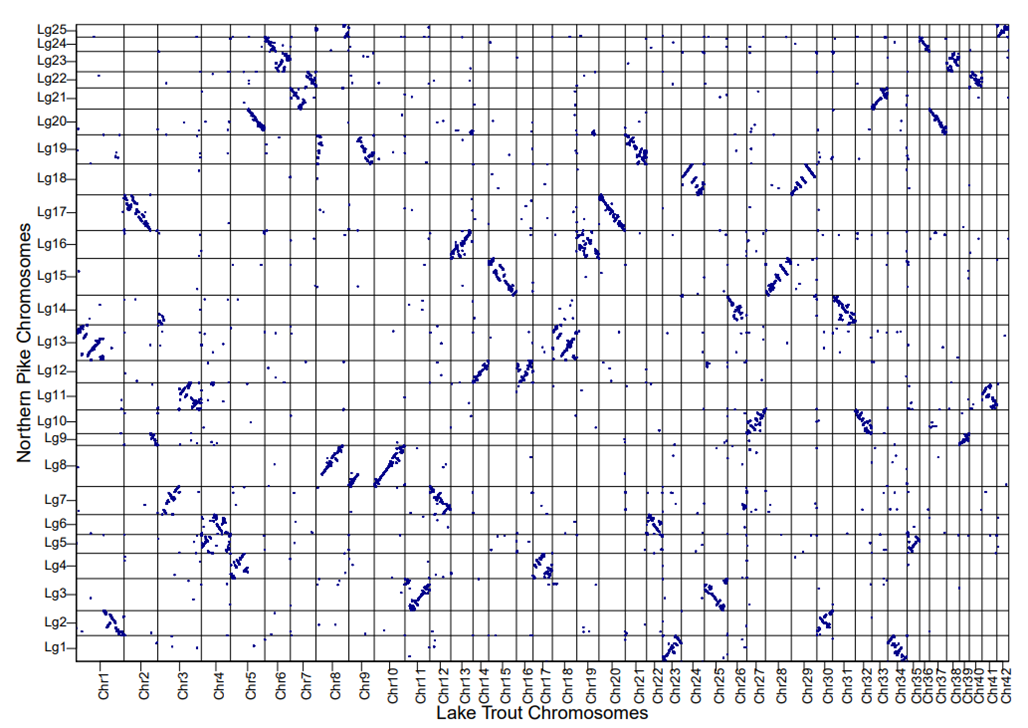


Supplemental Figure 6: Neighbor Joining Tree


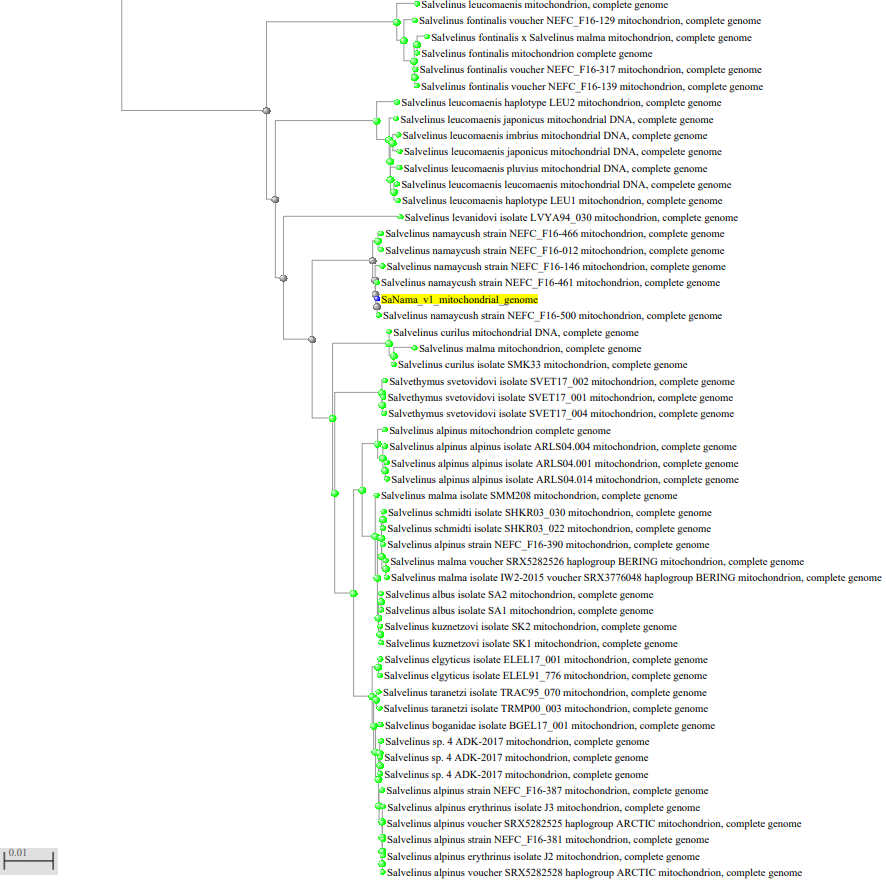
Neighbor joining tree comparing the Lake Trout mitochondrial genome assembly with blast hits in the NCBI nucleotide collection. The sequence assembled here is highlighted in yellow.

Supplemental Table 2: Comparison of BUSCO scores among salmonid genomes

Supplemental Table 3: N50 Comparison between salmonid genomes

Supplemental Table 4: Centromere Locations

Mean, median, minimum and maximum mapping positions for centromere associated RAD loci from the Smith et al. (2020) linkage map.
